# Common functional mechanisms underlying dynamic brain network changes across five general anesthetics: A rat fMRI study

**DOI:** 10.1111/cns.14866

**Published:** 2024-07-16

**Authors:** Sifan Chen, Bo Li, Ying Hu, Yizhe Zhang, Wanbing Dai, Xiao Zhang, Yan Zhou, Diansan Su

**Affiliations:** ^1^ Department of Anesthesiology, Renji Hospital School of Medicine, Shanghai Jiao Tong University Shanghai China; ^2^ Key Laboratory of Anesthesiology (Shanghai Jiao Tong University), Ministry of Education Shanghai China; ^3^ Department of Radiology The First Affiliated Hospital of Chongqing Medical University Chongqing China; ^4^ Department of Radiology, Renji Hospital School of Medicine, Shanghai Jiao Tong University Shanghai China

**Keywords:** concordance, dynamic graph theory, fMRI, functional connectivity, general anesthesia, rat, stability

## Abstract

**Background:**

Reversible loss of consciousness is the primary therapeutic endpoint of general anesthesia; however, the drug‐invariant mechanisms underlying anesthetic‐induced unconsciousness are still unclear. This study aimed to investigate the static, dynamic, topological and organizational changes in functional brain network induced by five clinically‐used general anesthetics in the rat brain.

**Method:**

Male Sprague–Dawley rats (*n* = 57) were randomly allocated to received propofol, isoflurane, ketamine, dexmedetomidine, or combined isoflurane plus dexmedetomidine anesthesia. Resting‐state functional magnetic resonance images were acquired under general anesthesia and analyzed for changes in dynamic functional brain networks compared to the awake state.

**Results:**

Different general anesthetics induced distinct patterns of functional connectivity inhibition within brain‐wide networks, resulting in multi‐level network reorganization primarily by impairing the functional connectivity of cortico‐subcortical networks as well as by reducing information transmission capacity, intrinsic connectivity, and network architecture stability of subcortical regions. Conversely, functional connectivity and topological properties were preserved within cortico‐cortical networks, albeit with fewer dynamic fluctuations under general anesthesia.

**Conclusions:**

Our findings highlighted the effects of different general anesthetics on functional brain network reorganization, which might shed light on the drug‐invariant mechanism of anesthetic‐induced unconsciousness.

## Graphical abstract

We found that the disruption of functional connectivity between subcortical and cortical regions by general anesthesia is a shared functional mechanism of various anesthetics. Moreover, the increased network and functional properties of higher‐order and unimodal cortical regions imply that the information flow required for consciousness is impaired within and between cortical and subcortical regions.

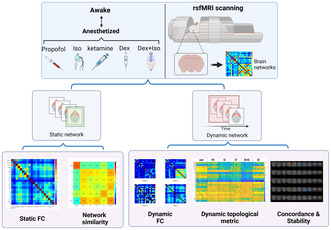

## 1 INTRODUCTION

Clinically, reversible loss of consciousness is the major therapeutic endpoint of general anesthesia. Different general anesthetic drugs, such as intravenous anesthetics like propofol, ketamine and dexmedetomidine, and volatile anesthetics like isoflurane, each with different molecular mechanisms and neuroanatomical basis, can all induce loss of consciousness.^1^, ^2^ Nevertheless, the drug‐invariant functional mechanism of anesthetic‐induced unconsciousness is still unclear.

General anesthetics have remarkably widespread effects on the brain function and network organization, and different theories of consciousness have emphasized different aspects of anesthetic effects on functional brain network.^3^, ^4^ The global neuronal workspace (GNW) theory posits that general anesthetics impair cortico‐cortical information transmission by altering the functional connectivity (FC) among key GNW components,^5^, ^6^ while other theories suggest that dysconnection within the thalamocortical network is the critical process leading to unconsciousness.^7^, ^8^ Recent dendritic information theory of consciousness proposed a new cellular‐level integration mechanism in which cortical layer 5 pyramidal neurons act as gates that modulate the global propagation of input information from higher‐order thalmamic areas and thus alter cortico‐cortical communication.^9^, ^10^ Another long‐help hypothesis proposes that subcortical brain regions implicated in sleep‐arousal regulation are key structures in anesthesia‐induced loss of consciousness.^11^, ^12^ Jointly, theoretical and experimental studies suggested that general anesthetics induce loss of consciousness via disruption of both cortico‐cortical and cortico‐subcortical communication. However, these models do not address how complex network interactions among distributed brain regions can be disrupted by chemically diverse anesthetics.

To identify potential drug‐invariant mechanisms of general anesthesia, here we conducted rest state functional magnetic resonance (rs‐fMRI) imaging on rat under five common used clinical anesthetics: propofol (PRO), isoflurane (ISO), ketamine (KET), dexmedetomidine (DEX) and combined isoflurane plus dexmedetomidine (ISO‐DEX). We then measured dynamic changes in FC, topological property, intrinsic brain activity, and functional architecture stability to identify brain regions with consistent alterations in response to all five agents.

## 2 METHODS

### 2.1 Animals

Male Sprague–Dawley rats (*n* = 57, 8–10 weeks old, 180–250 g) were accquired from Shanghai Sippr‐BK Laboratory Animal Co. Ltd. (China). The animals were housed under a 12/12 h light–dark cycle at 22 ± 2°C with 50%–60% humidity. Food and water were available ad libitum. All animal procedures used in this study were approved by the Animal Ethics Committee of Renji Hospital (RJ2022‐0730) and all methods were conducted in accordance with the related guidelines and regulations.

### 2.2 Administration of general anesthesia

Rats were randomly allocated to propofol group (PRO, *n* = 12; 10 mg/kg i.v. bolus and 60 mg/kg/h i.v.), isoflurane group (ISO, *n* = 13; 2.5%), ketamine group (KET, *n* = 11; 100 mg/kg i.p.), dexmedetomidine group (DEX, *n* = 10; 0.1 mg/kg i.v. bolus and 0.05 mg/kg/h i.v.) and combined isoflurane plus dexmedetomidine group (ISO‐DEX, *n* = 11; 0.05 mg/kg/h i.v. dexmedetomidine and 1% isoflurane). Briefly, all rats were initially anesthetized with ISO (3.5% induction and 2.5% maintenance) in a N_2_/O_2_ 70/30 mixture. Small cannulas were inserted into the tail vein of rats in groups that required continuous intravenous infusion of anesthetics (all excepted the ISO and KET group) and connected to a syringe pump (B. Braun, Germany). Isoflurane was discontinued after the surgical preparation and rats were then positioned in the scanning cradle. During the scanning setup procedure, rats awoke at the time‐point of approximately 10–15 min with close monitoring of respiration rate, and then the anesthesia switched to the tested anesthetic using the above‐mentioned protocol. During the anesthesia maintenance, the rats were given 1 L/min oxygen via a mask around the nose and a water‐circulated heating pad (TP500; Gaymar Industries, Orchard Park, NY) was used to maintain the body temperature. Heart rate (HR), respiratory rate (RR) and body temperature were continuously monitored during the fMRI scanning using the MR compatible small animal monitoring and gating system (SA Instruments, Stoney Brook, NY). The physiological data in all anesthesia groups were within the normal range during imaging acquisition (temperature: 37 ± 0.5°C; HR: 270–410 beats/min, RR: 60–90 breath/min, regular and no continuously rising for at least 2 min).

### 2.3 fMRI scanning

All fMRI scannings were performed on a 7 T Bruker 70/30 BioSpec with ParaVision 5.1 software (Bruker Biospin) and the rats were placed in a standard Bruker rat head coil. Functional Blood Oxygen Level‐Dependent (BOLD) images were acquired using single‐shot gradient‐echo echo‐planar imaging sequence (GE‐EPI) with the following parameters: repetition time (TR) = 1000 ms; echo time (TE) = 15 ms; slice number = 20; matrix size = 72 × 72; field of view (FOV) = 2.56 × 2.56 cm^2^; slice thickness = 2 mm; slice gap = 0 mm; 500 volumes per scan. High‐resolution anatomical images were acquired for each animal using fast spin‐echo sequence (TurboRARE) with the following parameters: TR = 3242 ms; TE = 37 ms; slice number = 35; matrix size = 350 × 350; FOV = 3.5 × 3.5 cm^2^; slice thickness = 0.6 mm; slice gap = 0 mm. The awake rat rs‐fMRI data in this study were acquired from an open database from Liu et al.,^13^ which used the same fMRI equipment and scanning parameters similar to those employed for our measurements.

### 2.4 Data preprocessing

**FIGURE 1:**
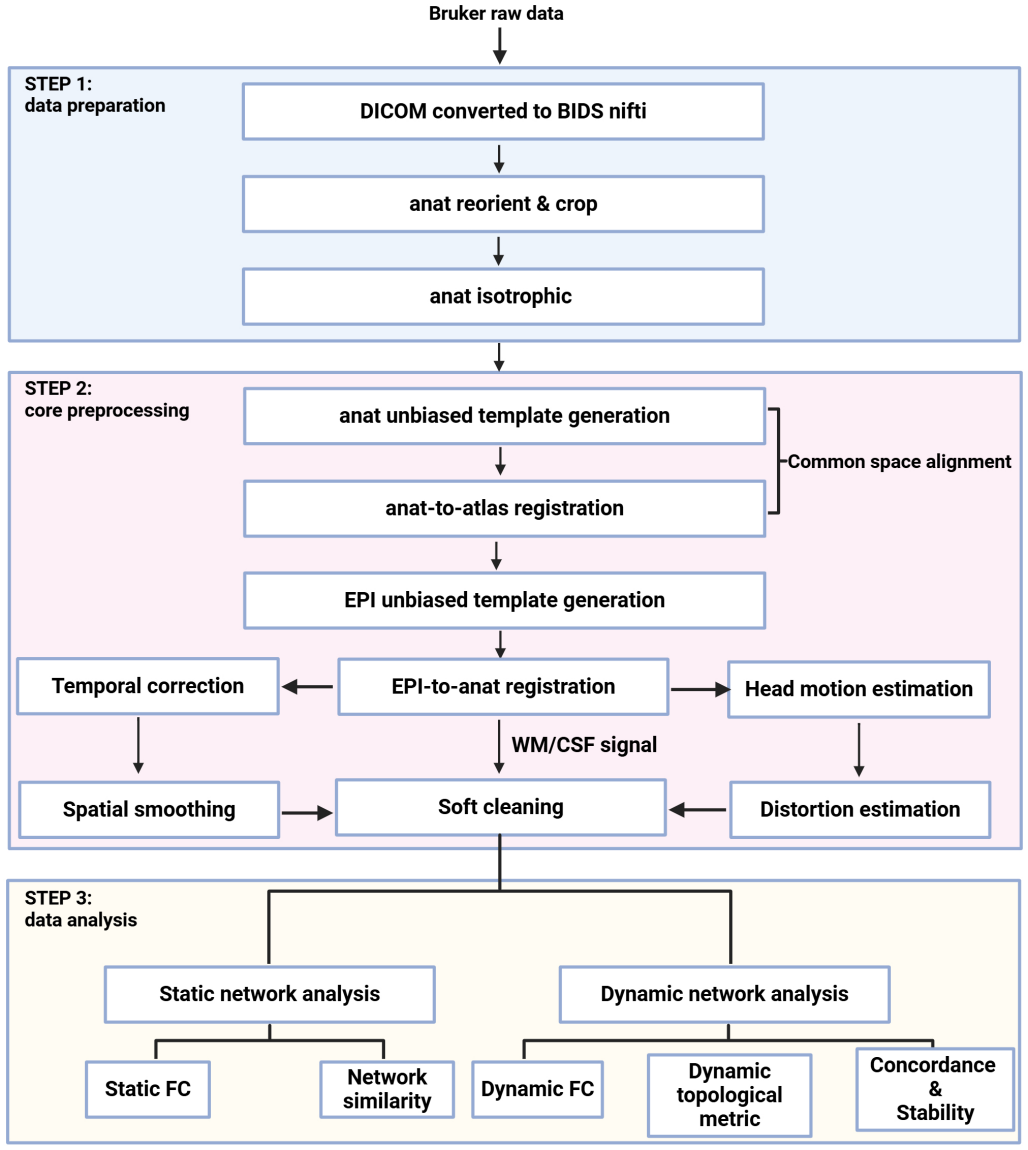
rsfMRI data preprocessing and analysis pipeline.

The data preprocessing procedures for both anesthetized and awake rats followed the guidelines given by StandardRat^14^ and the preprocessing pipeline of the recommended toolkit RABIES 0.4.6^15^ (https://github.com/CoBrALab/RABIES). The preprocessing procedures were summarized in Figure 1 and as follows:

1. Raw neuroimaging data were firstly converted from DICOM format into Nifti format using DPABI toolbox,^16^ and then the first 10 volumes of each animal were removed to ensure the stable magnetization and data organization according to the BIDS format. Second, T2‐weighted anatomical images were reoriented and cropped using the Reorient platform (https://neuroanatomy.github.io/reorient/) to improve the accuracy of atlas co‐registration. Finally, anatomical image resolution was adjusted to match that of the standard rat brain atlas (0.1 × 0.1 × 0.1 mm^3^) using MRIcroGL software (https://github.com/rordenlab/MRIcroGL).
2. Next, all T2‐weighted anatomical images within the same treatment group were iteratively generated to produce a dataset‐specific unbiased template. For this process, sequential images were registered to the average image generated by the overlap of all scans in the previous iteration to yield registrations of increasing stringency. Registration was performed twice per iteration to generate rigid and affine templates. A separate nonlinear transformation was performed in the last iteration to register each scan with an unbiased template. Finally, after the unbiased template was generated, Advanced Normalization Tools (ANTS, http://picsl.upenn.edu/software/ants/) was used to nonlinearly register the template with the preinput standard rat brain atlas.
3. Templates were then constructed for the functional EPI images using the same procedures. First, unbiased templates were generated and then registered with the anatomical images coregistered with the standard rat brain atlas in the previous step.
4. Temporal spikes in each voxel were removed using the 3dDespikes function in Analysis of Functional NeuroImages (AFNI).^17^ The average pruning value of all EPI images in a single scan was then calculated to generate a volume EPI image. This volume EPI image was used as the target, and head movement parameters were calculated by realigning each EPI frame to the target by rigid matching, thereby reducing functional image mismatch. Frames larger than the head movement amplitude threshold (0.15 mm) were excluded. Realignment was performed on EPI frames smaller than the threshold, and spatial distribution images of single‐voxel signal variation and time signal‐to‐noise ratio (tSNR) were calculated (Figure S1).
5. After computing the transformation needed to correct for head motion and magnetization distortion, slice timing correction was applied to the time series using the 3dTshift function in AFNI. Preprocessed EPI time series in local space were then generated by applying the transformation in a single re‐sampling operation for each EPI frame (avoiding multiple re‐sampling). Cascade transformation to make reference to the atlas for re‐sampling and generate a public space for preprocessing time series.
6. Finally, to minimize possible confounding information before data analysis, first‐order drift and mean signals were removed by voxel linear detrended processing. The mean signals within the white matter (WM) and cerebrospinal fluid (CSF) masks and the six head motion parameters were then regressed out for each voxel. This process did not include the average global brain signals from the regression because these contain important neurophysiological information and many studies have questioned the influence of this regression on dynamic FC analysis of spatial heterogeneity.^18^ Finally, the data were filtered in the time domain from 0.01 to 0.10 Hz and regional homogeneity (ReHo) calculated. After this calculation, images were then spatially smoothed using a 0.7‐mm full‐width at half‐maximum (FWHM) Gaussian filter (nilearning.image.smooth_img) to improve the SNR and eliminate artifacts.

### 2.5 Data analysis

#### 2.5.1 Static functional connectivity and matrix similarity analysis

The following 10 subcortical areas and 10 cortical areas were chosen as regions of interest (ROIs) that were previously identified to be involved in general anesthesia from the Waxholm Space Atlas,^19^ the standard brain template of Sprague–Dawley rats, using 3D Slicer software (Figure S2): higher‐order thalamus (Thal‐HO, including posterior complex, mediodorsal nucleus, anterodorsal nucleus, laterodorsal nucleus, central lateral nucleus, and posterior intralaminar nucleus), epithalamus (Thal‐EPI, including lateral habenular nucleus, medial habenular nucleus, nucleus of the stria medullaris, and pineal gland), nonspecific thalamus (Thal‐NS), hippocampus (Hippo), striatum (Stria), hypothalamus (Hypo), globus pallidus (Pal), basal forebrain (BF), reticular thalamic nucleus (TRN), ventral tegmental nucleus (VTA), cingulate cortex (Ccx), retrosplenial cortex (RSP), posterior parietal cortex (PPC), temporal association cortex(TC), orbital cortex (OC), somatosensory cortex (SS), visual cortex (VC), motor cortex (MC), insular cortex (Insular), and prelimbic cortex (PrL). Each ROI was made into a brain region mask, and the average time series of each ROI was extracted from all rats using DPABI software. The correlations between the average time series of each ROI pair were then calculated Pearson's correlation coefficients, yielding a 20 × 20 FC matrix for every rat. Each value in the matrix was then normalized by Fisher's Z transformation.

Statistical analyses between groups were conducted using Network‐based Statistics software.^20^ The static FC matrices of each anesthetic group and an awake control group were compared by independent samples *t*‐test with false discovery rate (FDR) correction to reduce type I error. An FDR‐corrected *p* < 0.05 was considered statistically significant. To further explore the similarity of the static FC network across individuals and groups^21^ (anesthetic and awake state groups), we linearized the original individual FC matrices using the internal function squeeze in Matlab, and calculated Pearson correlation coefficients between ROIs, yielding separate individual‐level similarity matrices for all ROIs, cortical ROIs, subcortical ROIs and cortico‐subcortical ROIs. Group averaged FC matrices were similarly analyzed to yield corresponding group‐level similarity matrices for all ROIs, cortical ROIs, subcortical ROIs and cortico‐subcortical ROIs.

#### 2.5.2 Dynamic functional connectivity and clustering analysis

The steps for dynamic FC analysis were similar to those for static FC analysis except that FC values in ROIs were extracted from the BOLD signal time domain using sliding window method. At present, there is no consensus on the optimal window width and step length, but it is generally believed that a time window of 30–60 s can capture brain activity dynamics as measured by BOLD signals with reasonable accuracy. According to previous studies, the window width was set to 50 s (50 TRs) and the step length to 1/10 the window width (5 s, 5 TRs).^22^ The average time series of each ROI in all rats was analyzed using the Matlab toolkit DynamicBC,^23^ yielding 89 × 20 × 20 FC matrices. Unsupervised k‐means clustering was performed on all FC matrices (including matrices of awake group and the five anesthesia groups) with the cluster number K value set to 4 according to the Davies–Bouldin Index (DBI). The final outputs were then used to construct distribution matrices of FC patterns at different times for calculation of FC pattern frequencies, group‐level statistical differences, and FC mode transition probabilities. Inter‐group comparison of FC pattern frequencies was conducted using one‐way analysis of variance (ANOVA) with post hoc Tukey–Kramer test (Graphpad Prism 9.5.1; GraphPad Software Inc., La Jolla, CA, USA) after checking normality and homoscedasticity with Shapiro–Wilk test and Bartlett's test. *p* < 0.05 was considered statistically significant.

### 2.6 Graph theory analysis

In this study, ROIs in FC matrices were defined as network nodes, and the absolute FC strength values after Fisher Z transformation were converted to edges. The threshold sparsity degree S of each binary network was set to 0.2 based on previous studies^24^ so that only the strongest 20% of connections were retained (weaker connections were set to 0), thereby eliminating spurious edges caused by physiological noise and other factors. The Gretna toolkit^25^ running in Matlab was used to calculate network topological indicators of dFC matrices obtained from each rat in the previous step. The topological indicators derived from this analysis were nodal efficiency, nodal clustering coefficient, and nodal shortest path length.

### 2.7 Dynamic intrinsic brain indicators

#### 2.7.1 Concordance

Concordance was calculated via Temporal Dynamic Analysis, which is an extension function of DPABI toolbox. In this analysis, we derived five common indices of spontaneous brain activity, including fractional amplitude of low‐frequency fluctuations (fALFF), regional homogeneity (ReHo), voxel mirror homotopic connectivity (VMHC), degree centrality, and global signal correlation (GScorr).

1. Low‐frequency oscillation amplitude is susceptible to noise and is strongly associated with fALFF, so only fALFF was calculated. A low‐frequency amplitude score was calculated for each frequency amplitude in the range 0.01–0.10 Hz by Fourier analysis and normalized to the sum of the entire frequency range for each ROI as a measure of local brain activity.
2. Regional homogeneity is defined as the correlation between the BOLD time series of a given voxel and its neighbors 26 voxels and the distribution is expressed as the Kendall coefficient to indicate the degree of synchrony across brain regions.
3. Voxel mirror homotopic connectivity is defined as the BOLD time series correlation (Pearson's *r*) between each homotopic pair of voxels in left and right hemispheres. The Fisher Z‐transform values were calculated as measures of inter‐hemispheric communication.
4. Degree centrality was calculated as the weighted sum of positive correlations for each voxel with r values greater than 0.25 and *p* < 0.05.
5. Global signal correlation (GScorr) was defined as the Fisher's Z‐transformed Pearson's correlation coefficients between each voxel and the mean BOLD time series in the mask (in this case, the whole‐brain mask).

First, voxel‐wise concordance of BOLD signals was calculated at the voxel level with a sliding window width of 50 s and step size of 5 s, and the five brain spontaneous activity indicators were calculated for each voxel in each time window. This analysis was then repeated at the volume level. The Kendall's W of each time window was then calculated to represent dynamic concordance among voxels. These values were then Fisher's Z‐transformed to obtain output maps of voxel‐level concordance. Then, concordance was calculated across all brain voxels for each participant (volume‐wise concordance).

#### 2.7.2 Stability

The Stability of dynamic functional brain networks was calculated using Stability Analysis of DPABI.^26^ For all voxels in the brain, dynamic functional structure stability of dynamic FC between a single voxel and all other voxels in the mask (in this case, the gray matter mask) was calculated using Kendall's W. Specifically, a sliding window (with the same width and step size as in previous sections) was used to the calculate voxel‐wise FC values within each successive window (89 in total), and the voxel‐level FC was then also Fisher's Z‐transformed to determine the functional structure of each voxel.

## 3 RESULTS

### 3.1 Distinct static FC networks induced by different anesthetics

**FIGURE 2:**
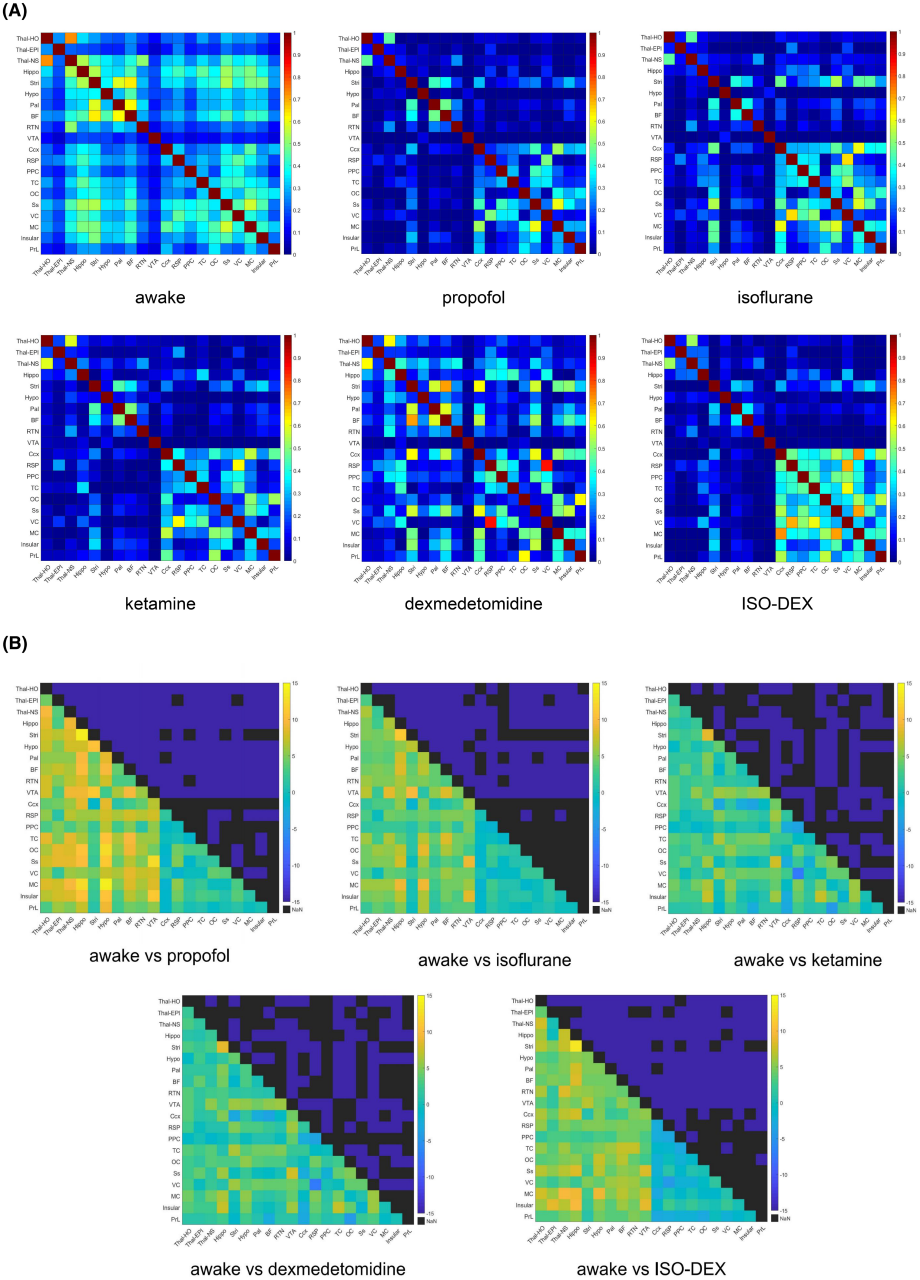
Comparison of static FC in awake state and different anesthetized states. (A) Averaged connectivity matrices in awake, propofol anesthetized, Isoflurane anesthetized, ketamine anesthetized, dexmedetomidine anesthetized and ISO‐DEX anesthetized conditions. (B) Comparison of each ROI‐ROI correlation coefficient between awake and anesthetized groups. The lower left shows the T values, and the black boxes in the upper right indicate the significant differences (*p* < 0.05, FDR‐corrected paired *t*‐test). BF, basal forebrain; Ccx, cingulate cortex; Hippo, hippocampus; Hypo, hypothalamus; Insular, insular cortex; MC, motor cortex; OC, orbital cortex; Pal, pallidum; PPC, posterior parietal cortex; PrL, Prelimbic area; RSP, retrosplenial cortex; RTN, reticular thalamic nucleus; Ss, somatosensory cortex; Stri, striatum; TC, temporal association cortex; Thal‐EPI, epithalamus; Thal‐HO, higher order thalamus; Thal‐NS, nonspecific thalamus; VC, visual cortex; VTA, ventral tegmental area.

We firstly investigated the static brain ROI FC under five anesthetics and awake conditions. The results suggested that each anesthetic induced different static FC network (Figure 2A). All five anesthetics induced widespread reductions in FC strength among various subcortico‐subcortical and subcortico‐cortical networks compared to the awake state (Figure 2B, *p* < 0.05, *t*‐test, false discovery rate [FDR] corrected). In contrast, FC strength in cortical–cortical networks did not differ significantly between awake and most anesthesia conditions. In the dexmetatomidine group, however, the static FC pattern differed from other anesthetics in that FC strengths were widely reduced compared to the awake state and statistical differences were relatively weak in all cortico‐cortical, subcortico‐cortical, and subcortico‐subcortical ROI pairs.

### 3.2 Effects of different anesthetics on the similarity of static FC

**FIGURE 3:**
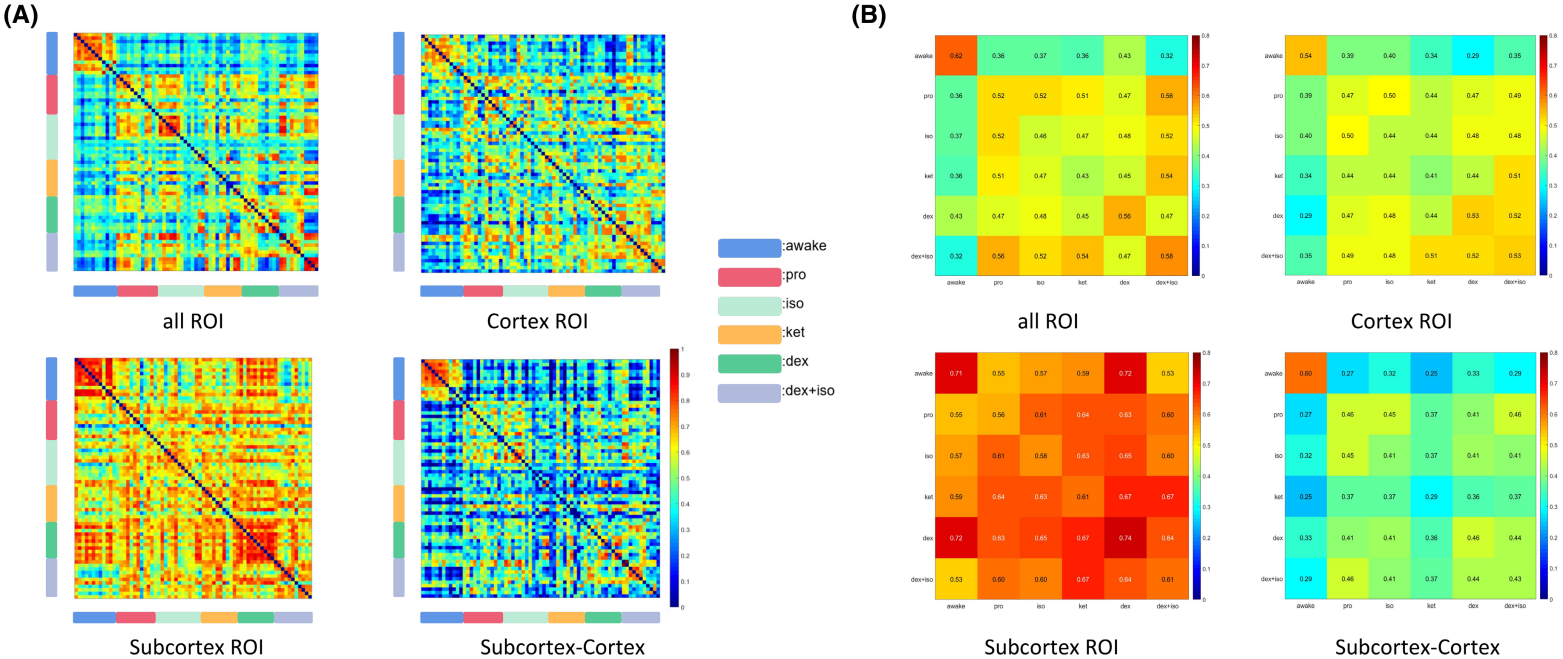
The similarity of functional brain connectivity among awake and anesthetized groups. A similarity matrix, where every cell represents the similarity of functional networks among (A) individual and (B) group levels. Rows and columns represent the similarity between a particular individual and all individuals, including other groups. The similarity matrix comprises the awake group, propofol group, isoflurane group, ketamine group, dexmedetomidine group and Iso + dex group. Color bar, correlation coefficient.

Then we conducted similarity analysis of static FC matrices at the individual and group levels (Figure 3). Under awake conditions, substantial similarity was observed in individual FC matrices of all ROIs (mean:*z*(*r*) = 0.79), cortical ROIs only (mean:*z*(*r*) = 0.66), subcortical ROIs only (mean:*z*(*r*) = 0.99), and from cortical–subcortical ROI pairs (mean:*z*(*r*) = 0.65), indicating that the functional networks in the awake state are robust with stable individual features (Figure 3A). In contrast, networks constructed from all ROIs, cortical ROIs only, and between cortico‐subcortical ROI pairs of rats under anesthesia differed significantly from those in the awake state. Notably, cortical–subcortical networks showed the lowest similarity, with mean *z*(*r*) ranging from 0.16 to 0.40, suggesting that modulation of FC strength within cortico‐subcortical networks may be the primary mode of action shared by different general anesthetic drugs. In contrast, the similarity was unexpectedly high within subcortical networks, with mean *z*(*r*) values ranging from 0.61 to 0.87 and the highest values in dexmedetomidine group (mean:*z*(*r*) = 0.87) (Figure 3A). Networks from all five anesthetic groups showed moderately but still significantly lower similarity values than the awake group, indicating that different general anesthetics can substantially modify functional network structures (Figure 3B). Jointly, these findings suggest that functional networks are significantly modified by general anesthetics that different anesthetics have distinct effects on the static FC networks, and that the anesthetic‐dependent variation is maximal within the cortical–subcortical networks.

### 3.3 Dynamic FC patterns during different anesthetics

**FIGURE 4:**
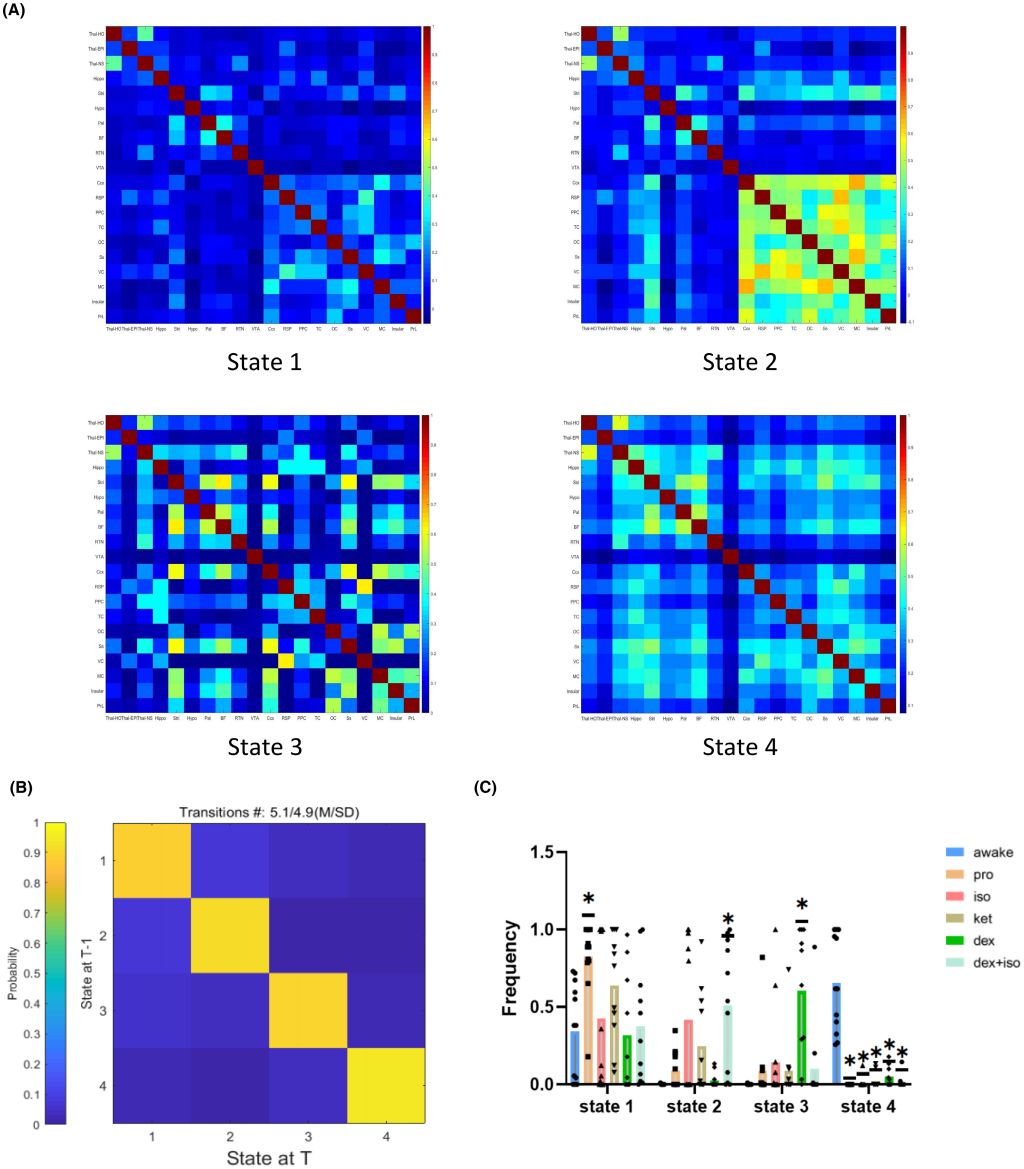
Dynamic functional connectivity in awake and propofol, isoflurane, ketamine, dexmedetomidine and iso‐dex anesthetized mice. (A) Four dynamic FC states. (B, C) Transition probablity between states (B) and frequency of occurrence (C) in each state in the awake condition and different anesthetized states. *p < 0.05 compared with the awake condition in each state, Tukey–Kramer test. BF, basal forebrain; Ccx, cingulate cortex; Hippo, hippocampus; Hypo, hypothalamus; Insular, insular cortex; MC, motor cortex; OC, orbital cortex; Pal, pallidum; PPC, posterior parietal cortex; PrL, Prelimbic area; RSP, retrosplenial cortex; RTN, reticular thalamic nucleus; Ss, somatosensory cortex; Stri, striatum; TC, temporal association cortex; Thal‐EPI, epithalamus; Thal‐HO, higher order thalamus; Thal‐NS, nonspecific thalamus; VC, visual cortex; VTA, ventral tegmental area.

We next investigated the time‐varying FC patterns and spatiotemporal dynamics of the five anesthetics. Using the data‐driven k‐means clustering (sqEuclidean distance, *k* = 4), we partitioned the windowed FC matrices in the awake state and the five anesthetic regimens across all animals into four distinguishable connectivity patterns (Figure 4A). State 1, characterized as overall weak FC, was present almost exclusively under the five anesthetic regimens. In sharp contrast, a highly complex FC pattern (State 4) with coordinated between‐network and within‐network FC was more prevalent in awake state. Besides, State 2, representing preserved cortical connectivity, were probable across all anesthetic groups but showed the highest occurrence in ISO, DEX + ISO group, and State 3, characterized by a relatively complex but suppressed brain‐wide connectivity occurred exclusively in DEX group (Figure 4C). Notably, the transition probability between FC patterns is extremely low, indicating that the brain‐wide inhibition of functional connectivity may be the basic feature of different anesthetics (Figure 4B).

### 3.4 Topological characteristics of the time‐varying FC networks under different anesthetics

**FIGURE 5:**
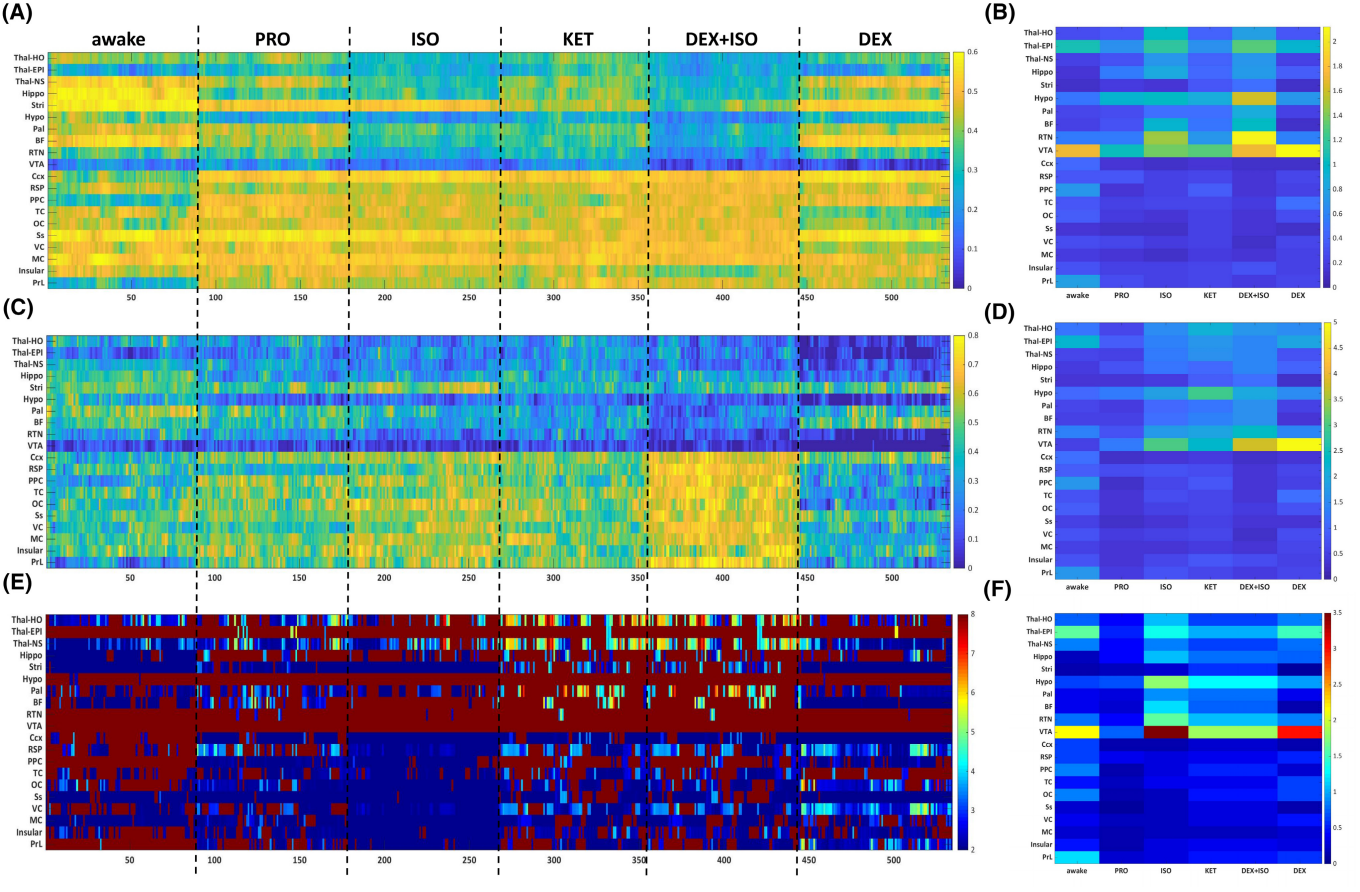
Time‐resolved analysis of regional topological characteristics in awake state and propofol, isoflurane, ketamine, dexmedetomidine and iso‐dex anesthetized state. (A, C, E) ROI‐Timepoint heatmap of nodal efficiency (A), nodal clustering coefficiency (C) and nodal shortest path (E). (B, D, F) average coefficient of variation (C.V.) of each topological indicator of ROI in each brain state. BF, basal forebrain; Ccx, cingulate cortex; Hippo, hippocampus; Hypo, hypothalamus; Insular, insular cortex; MC, motor cortex; OC, orbital cortex; Pal, pallidum; PPC, posterior parietal cortex; PrL, Prelimbic area; RSP, retrosplenial cortex; RTN, reticular thalamic nucleus; Ss, somatosensory cortex; Stri, striatum; TC, temporal association cortex; Thal‐EPI, epithalamus; Thal‐HO, higher order thalamus; Thal‐NS, nonspecific thalamus; VC, visual cortex; VTA, ventral tegmental area.

Furthermore, we investigated the topological characteristics of the time‐varying FC networks using graph‐theory and examined whether they could be used to describe the different anesthetic states. For this purpose, we introduced three nodal topological indexes to analyze the time‐varying FC matrices obtained in the previous step, aiming to characterize the dynamic evolution of the topological changes in brain‐wide networks (Figure 5) and cortical networks (Figure S3).

Time‐resolved analysis of nodal efficiency (Figure 5A) suggested that each anesthesia group maintained high nodal efficiency in cortical brain areas, and nodal efficiency was actually enhanced within important cortical regions such as cingulate, retrosplenial, prelimbic, and posterior parietal cortex. In contrast, nodal efficiency was reduced in subcortical regions such as the higher‐order thalamus, nonspecific thalamus, hippocampus, and hypothalamus by all anesthesia regimens compared to the awake state. The changes in time‐varying nodal characteristics were quantified by calculating the coefficient of variation (CV) at each brain nodes (Figure 5B). Notably, nodal efficiency in cortical brain regions showed lower CV across different anesthetics compared to the awake state, whereas subcortical brain regions demonstrated higher CV values in nodal efficiency under all anesthetics. Moreover, nodal efficiency analysis of cortical networks alone (Figure S3A) suggested that cingulate cortex and prelimbic cortex maintained greater nodal efficiency under all anesthesia regimens compared with the awake group, while nodal efficiency of the retrosplenial cortex, posterior parietal cortex, and insular cortex were lower than in the awake condition. Notably, unimodal cortical regions such as somatosensory, motor, and visual cortex still maintained high nodal efficiency under different anesthetics.

To further clarify the impact of varied general anesthetics on the information transmission within spatially distributed brain regions, we calculated the nodal clustering coefficient as an index of short‐range communication (Figure 5C) and the nodal shortest path as an index of long‐range transmission (Figure 5E). In awake state, low nodal shortest path values have been observed in subcortical regions such as the epithalamus, non‐specific thalamus, and hippocampus, indicating that these important subcortical regions can maintain efficient information exchange with other brain regions in the functional network. Conversely, under the action of different anesthetics, the nodal shortest path in subcortical brain area increased dramatically, while that in cortical brain area remains low, suggesting that the remote information transmission between subcortical brain area and other brain regions may be inhibited by general anesthesia, while the cortical brain areas is relatively unaffected (Figure 5E). A similar result was found when analyzing the nodal shortest path in the cortical network alone (Figure S3B). Excepted for a slight decrease in the cingulate area, values were similar to the awake state, which further suggests that anesthetics have relatively little effect on long‐range information transmission in cortical brain networks. Similarly, nodal clustering coefficient analysis indicated that short‐range information transmission among subcortical regions was severely inhibited by all 5 anesthetics (Figure 5C), while transmission among cortical networks was maintained or even slightly enhanced (Figure S3C). Consistent with nodal efficiency analysis, CVs of nodal clustering coefficients and nodal shortest paths among cortical regions were relatively low in anesthetized state, indicating fewer dynamic fluctuations in terms of long‐ and short‐range information transmission, whereas these CVs were high among subcortical brain regions (Figure 5D,F).

### 3.5 Voxel‐level time‐varying measures underlying intrinsic connectivity and the dynamic connectivity architecture of the anesthetized brain

Considering that the topological properties are abstract concepts, the calculation is based on the abstraction of each brain regions and functional connections into nodes and edges, while ignoring the existence of anatomical connections in any brain interval in the network. Therefore, we explored the effects of different anesthetics on the intrinsic connectivity and the dynamic connectivity architecture of the anesthetized brain in the time domain at the voxel level by examining two newly developed connectivity indicators, *Concordance* and *Stability*.

**FIGURE 6:**
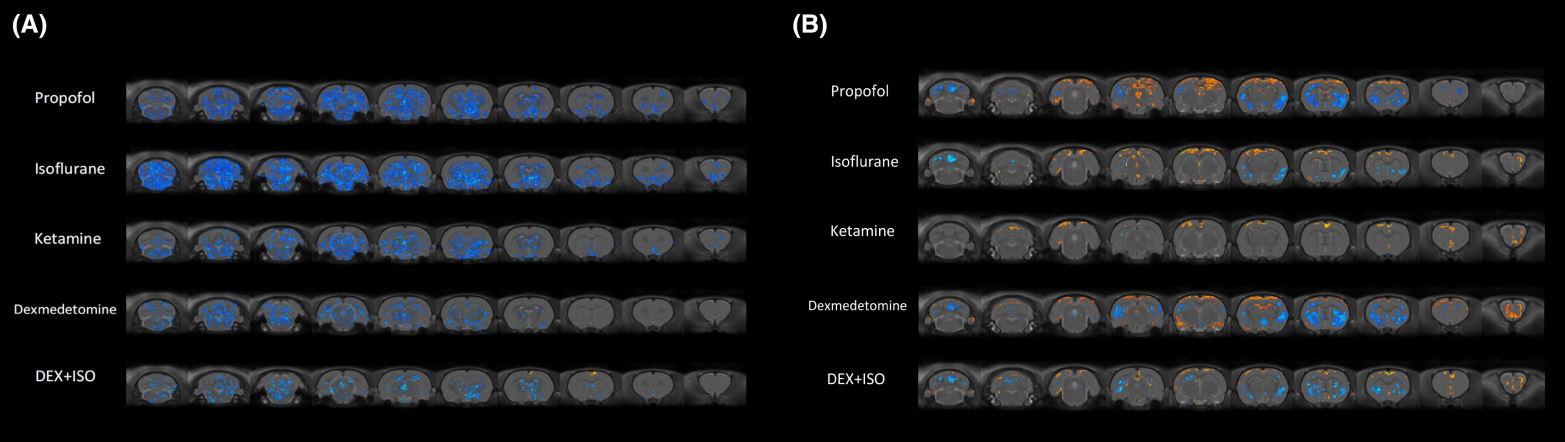
Voxel‐level analysis of intrinsic connectivity and dynamic connectivity architechture using Concordance (A) and Stability (B). (A) Significant increase in Concordance (hot color) and decrease (blue color) in the each anesthetized group compared with the awake group (*p* < 0.05, paired *t*‐test false discovery rate (FDR)‐corrected at voxel level). (A) Significant increase in Stability (hot color) and decrease (blue color) in the each anesthetized group compared with the awake group (*p* < 0.05, paired *t*‐test false discovery rate (FDR)‐corrected at voxel level).

Concordance was proposed by Yan et al.,^27^ which aims to compare and integrate multiple commonly used intrinsic brain function indices, such as fALFF, ReHo, DC, GSCorr, VMHC. Currently, this summary indicator has been studied in healthy subjects and patients with various mental and psychological diseases, and has been found to correlate strongly with different symptoms.^28^ However, the influence of general anesthetics on concordance has not been studied. Compared to the awake state, the whole‐brain volume‐based concordance was significantly reduced under general anesthesia, suggesting that general anesthetics reduce intrinsic connectivity at the whole‐brain level (Figure S4). Further voxel‐based concordance analysis showed that the brain regions with significantly reduced concordance compared to the awake state were mainly subcortical brain regions, while the concordance among cortical brain regions was relatively less affected, consistent with our results in dynamic topological analyses (Figure 6A).

Additionally, we also examined the whole‐brain spatiotemporal dynamics of FC architecture directly by calculating FC stability across the brain following exposure to different general anesthetics (Figure 6B). An interesting finding is that all general anesthetics tested tended to simultaneously increase the stability of functional communication among unimodal brain regions such as primary sensory and motor cortices and higher association regions such as cingulate cortex, while reducing FC stability in key subcortical brain regions. In accord with our initial results, the FC among cortico‐subcortical brain regions was significantly inhibited under different general anesthetics. We speculate that general anesthetics mediate the loss of consciousness by interrupting functional connections between subcortical and cortical brain areas, resulting in a highly unstable state and disrupting the transmission of peripheral information to the cortex.

## 4 DISCUSSION

The main finding of this study is that multiple chemically distinct general anesthetics induced distinct patterns of functional conncectivity inhibition within brain‐wide networks, and the cortical–subcortical network instead of the cortical network were preferentially disrupted. Dynamic information transmission capacity and intrinsic connectivity were also substantially reduced in subcortical brain regions but relatively preserved in cortical regions. Notably, the temporal variability in information exchange in unimodal and higher association cortices were specifically reduced under general anesthesia, suggesting that anesthetics impair their capacity to reorganize connectivity patterns. Together, this inflexibility combined with disruption of subcortical–cortical communication disrupts the transmission and processing of information in cortex and maintains loss of consciousness.

There have been long‐lasting controversies regarding the anesthetic effects on the cortico‐cortical network. Previous studies have reported that frontoparietal and default mode networks are disrupted by major classes of general anesthetics, suggesting common cortical network mechanisms, and more recent studies have also reported functional disconnection of the prefrontal cortex from posterior cortex under several general anesthetics.^29^, ^30^, ^31^, ^32^ However, Ma et al.^33^ showed that propofol enhanced the oculomotor circuit's neural coupling, contradicting the expected reduction of connectivity in the closely connected brain regions in fronto‐parietal network. Moreover, more recent neuroimaging studies using stereo‐EEG with high spatio‐temporal resolution suggested that the primary auditory area remains responsive to sounds under propofol anesthesia and propofol mainly targets the propagation of auditory activity between the cortical areas.^34^ Thus, it appears that general anesthetics do not uniformly reduce functional connectivity within cortico‐cortical networks, and that local brain networks remain intact. Rather, cortical network activity may be functionally isolated from subcortical activity under general anesthesia. Indeed, in the current study we found that cortical FC was largely preserved while cortical–subcortical FC was dramatically reduced under general anesthesia, indicating that functional disconnection in cortical regions might not be necessary for general anesthesia‐induced unresponsiveness.^35^ Thus, cortico‐cortical disconnection alone may not expect to be a reliable correlate of general anesthesia.

Emerging research acknowledges anesthesia as a dynamic process, contrasting with the previously considered static state; however, the precise effects of anesthetics on brain network dynamics remain largely unexplored.^32^, ^36^, ^37^ Here we observed that the patterns of brain‐wide inhibition of FC and preserved FC in cortico‐cortical networks (State 1&2) were increased in terms of occurrence frequency under different general anesthetics. Previous rodent neuroimaging studies compared the static FC between conscious and lightly anesthetized states, while they ignored the temporal dynamics in FC patterns transition and their ROI were too large to evaluate the subnucleus level.^38^, ^39^ Notably, a complex but overall inhibited FC pattern was specifically observed in the DEX group (State 3). We inferred that it is because dexmedetomidine has unique clinical properties compared to the other anesthetics by producing moderate sedation state similar to sleep.^40^ Conversely, a highly complex functional connectivity pattern (State 4) emerges exclusively in the conscious state, characterized by coordinated functional connection across cortical and subcortical regions, which represents the complex cooperative working state of brain regions. There was a very low transition probability among different FC patterns, indicating their distinct functional network characteristics. Notably, significant differences in FC networks were observed between the awake state and all anesthetized states, in both dynamic and static analysis. This observation undoubtedly implicates that the pharmacological mechanisms of one or a few anesthetics cannot be indiscriminately generalized to the broader spectrum of anesthetics. Particularly, those anesthetics with disparate molecular structures and neuroanatomical targets necessitate individualized research endeavors to elucidate their distinct effects comprehensively.

Although the topological properties of anesthetized brain network have been gaining increasing popularity, with several lines of evidence indicating that anesthetics tend to preferentially disrupt activities of hub structures, fragmenting hierarchical networks and disrupting the normal organization of functional brain networks, the temporal topology of anesthetized brain is still little studied.^4^ Interestingly, by introducing the time scale into the analysis of topological properties, we found that anesthetics did not substantially alter information exchange within cortical networks. Conversely, both long‐range and short‐range information transmission capacities were slightly increased in cortical regions across all anesthetic regimens except for dexmedetomidine, whereas the temporal variation in information exchange in all cortical regions were impaired. This topological reconfiguration in terms of the dynamic inflexibility in cortical network under general anesthesia may in turn disturb higher‐order information processing despite strong FC.^41^ Moreover, all anesthetics increased FC stability in high‐order association and unimodal cortical regions. High‐order cortices such as the cingulate and prelimbic cortex require high FC stability as they integrate multimodal information from other brain regions, whereas unimodal regions have lower FC stability as they must respond to changing external stimulus conditions and require top‐down regulation from high‐order areas for efficient processing of behaviorally relevant information.^42^, ^43^ These general anesthetics increased the FC stability of unimodal regions, which may impair sensory‐motor information processing and lead to functional separation from higher‐order association regions, thereby impairing consciousness.

These findings that general anesthetics preferentially disrupt network connections within subcortical regions and between cortical and subcortical regions are in accord with the findings of Suzuki et al.^9^ that isoflurane anesthesia downregulated higher‐order thalamic activity, which in turn inhibited thalamocortical signaling by uncoupling the distal dendrites and cell bodies of L5 pyramidal neurons. Additionally, central thalamus stimulation in anesthetized rodents and primates reversed the unconsciousness state and showed awake‐like neurobiological features.^44^, ^45^, ^46^ Anesthetics also reduced connectivity stability in key subcortical areas. We hypothesize that anesthetics disrupt FC and increase instability between subcortical and cortical regions, disrupting peripheral information transmission to cortical areas and causing loss of consciousness.^26^, ^47^ Notably, functional correlates of different conscious states including anesthesia and disorders of consciousness were supposed to be the basis to identify the recordable signatures of consciousness. In this study, we calculated the FC matrix similarity of each anesthetized state and awake state, and our data showed that cortico‐subcortical network had the consistent lowest similarity under different anesthetics, meanwhile the group levels of similarity in each anesthetic regimen were moderate (0.41–0.46) expect for ketamine, it is then speculated that it might be an easily recordable physiological marker to assess the anesthetic or conscious level. Altogether, these results might also advance the use of rs‐fMRI to detect the presence or absence of consciousness.

This study has some limitations that need to be addressed. Firstly, the relatively small sample size and scanning volumes limited the generalizability of our results to major surgical anesthesia as well as the exploration for the topological dynamics of different general anesthetics, especially the temporal state transitions. Subsequent research with larger sample sizes and data volumes is imperative to augment the extant comprehension of the intricate dynamics encompassing general anesthesia. Secondly, the neuroimaging data from awake rats were obtained from the open database by Liu et al., which may have introduced unknown bias, although these data were obtained using the same MRI scanner and similar acquisition protocol as this study. Notably, there are major challenges for awake rodent fMRI imaging because of the significant motion and stress during imaging acquisition and there have been controversies regarding the imaging protocols. Considering that the dataset from Liu et al. is one of the most influenced open database in rodents with rigorous standardization and validation, we believe that the integration of the Liu et al. dataset outweigh the potential drawbacks. Finally, analysis of voxel‐wise indicators in the subcortical regions were more easily affected by the noise than in the cortical regions. Although we have regressed out the signals from white matter and CSF, subsequent studies with neuroimaging techniques that directly record signals in subcortical regions, such as stereo‐electroencephalography (sEEG), may provide more accurate measures of subcortical network activity.

In summary, our results suggest that the disruption of functional connectivity between subcortical and cortical regions by general anesthesia is a shared functional mechanism of various anesthetics. Moreover, the increased network and functional properties of higher‐order and unimodal cortical regions imply that the information flow required for consciousness is impaired within and between cortical and subcortical regions.

## CONFLICT OF INTEREST STATEMENT

There is conflict interest.

## Supporting information


Figure S1.

Figure S2.

Figure S3.

Figure S4.

## Data Availability

The data that support the findings of this study are available from the corresponding author upon reasonable request.
