# Supplementary figures and images for: Common functional mechanisms underlying dynamic brain network changes across five general anesthetics: A rat fMRI study
Source: CNS Neurosci Ther. 2024 Jul 16;30(7):e14866. doi: 10.1111/cns.14866 (PMC11251872; doi:10.1111/cns.14866)

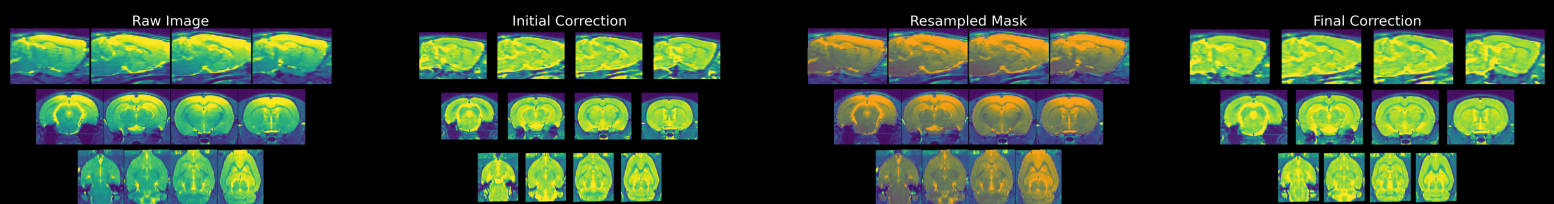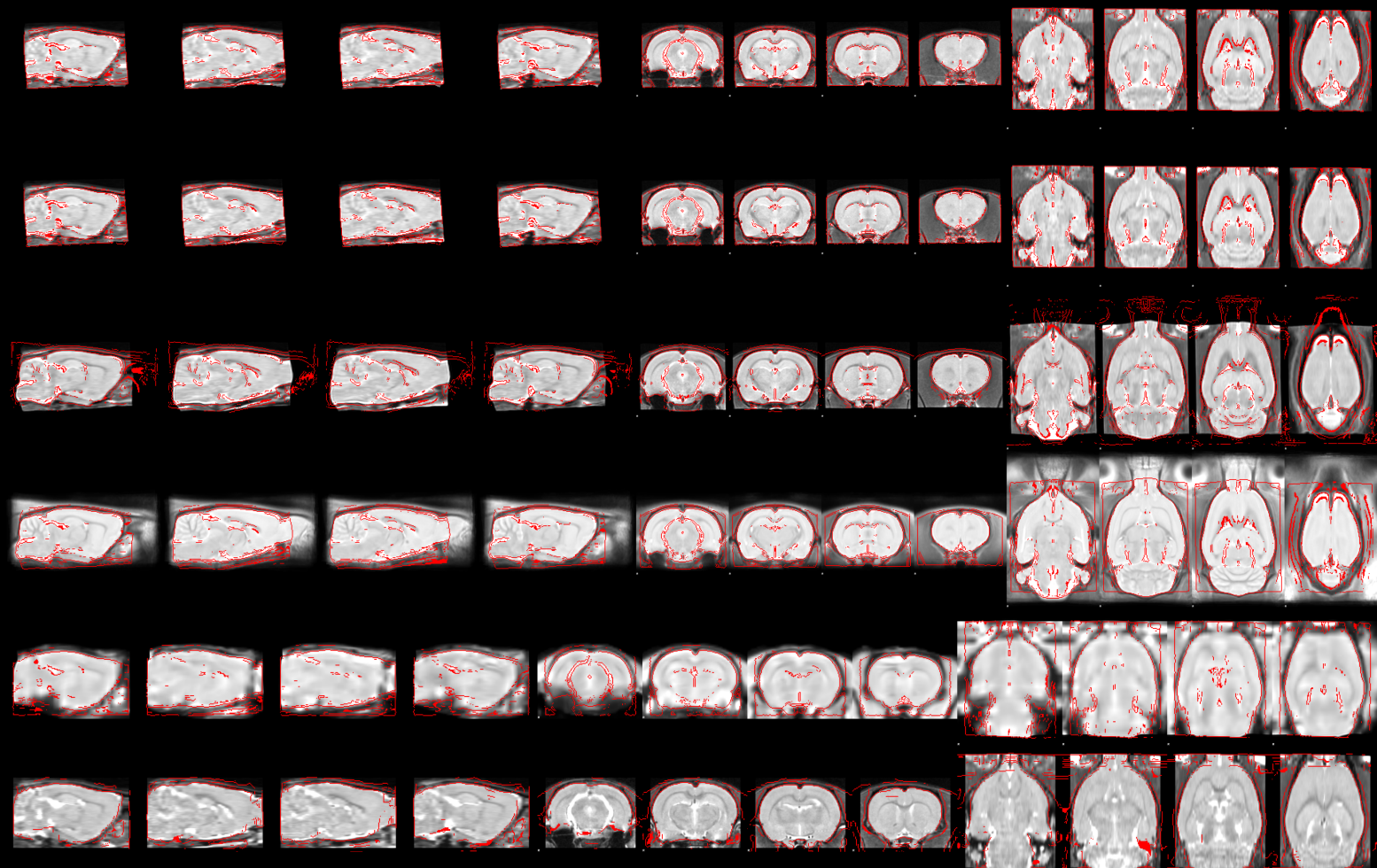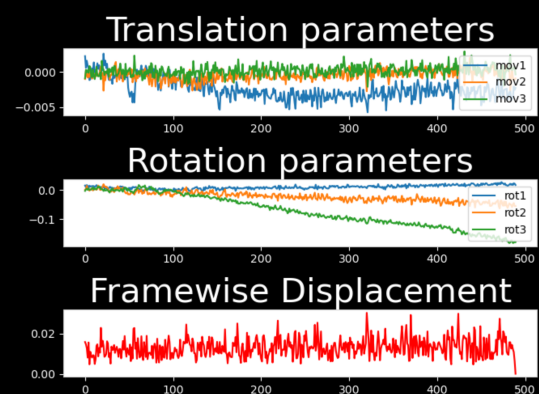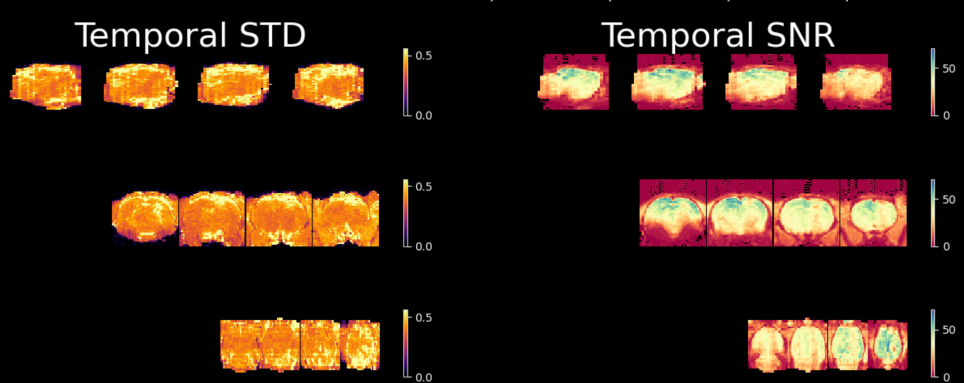

Supplement: Supplementary file 1 — Figure S1. [file CNS-30-e14866-s003.pdf]

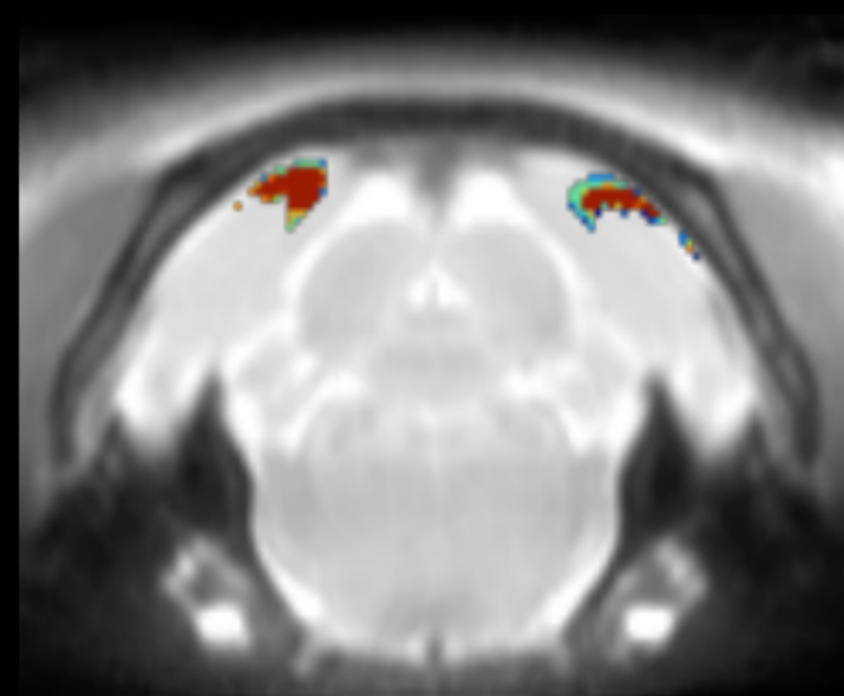

-8.8

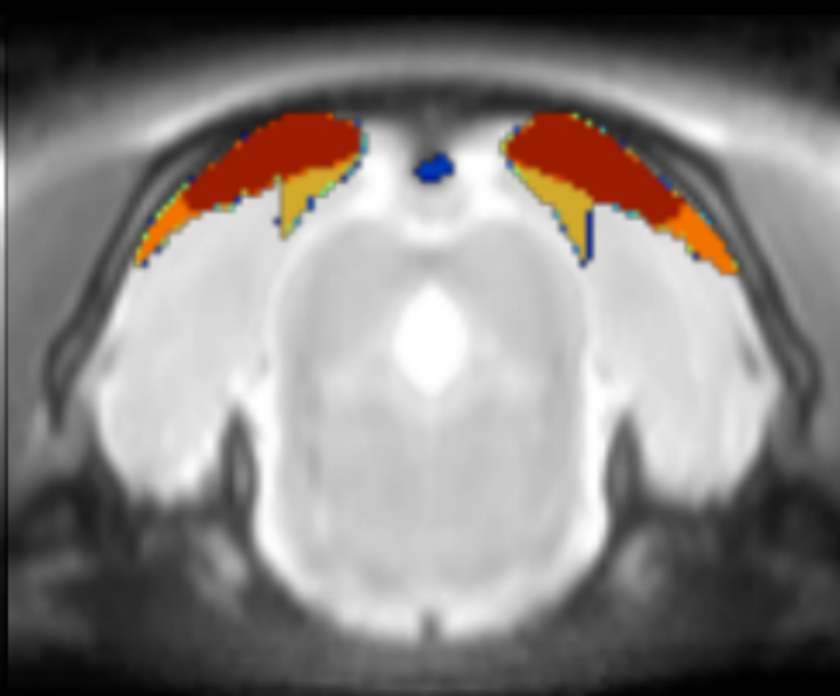

-8.1

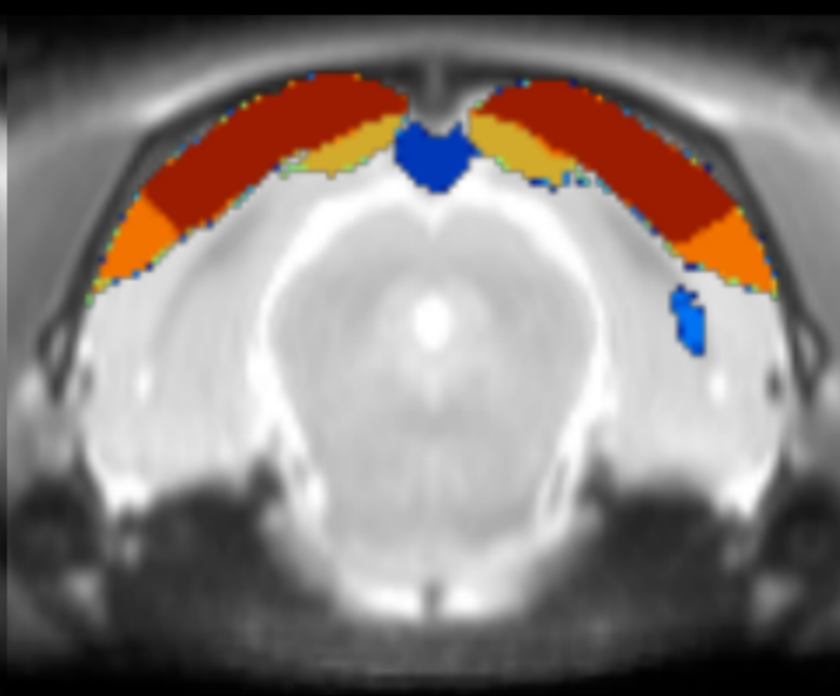

-7.1

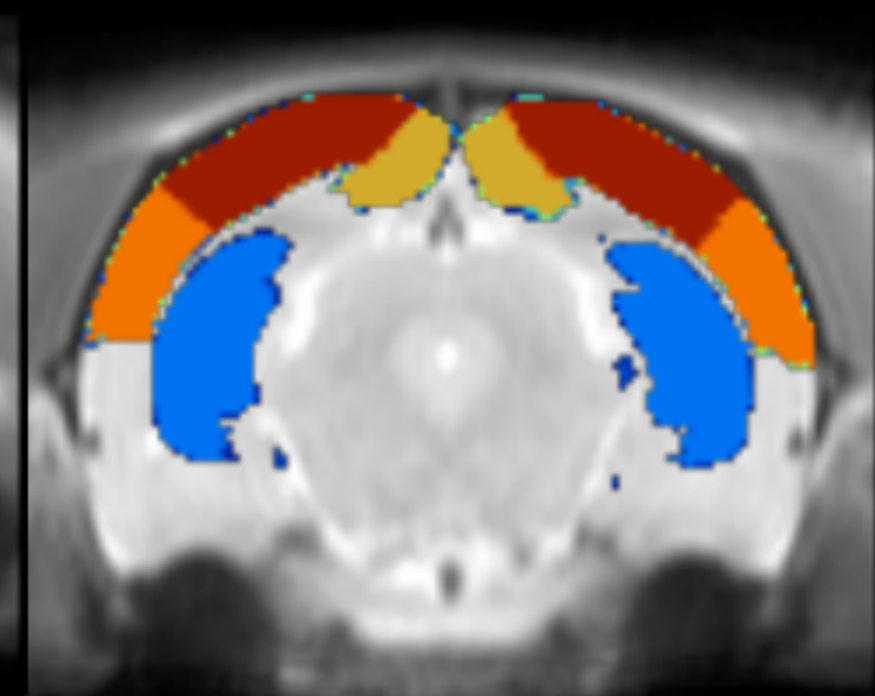

-6.1

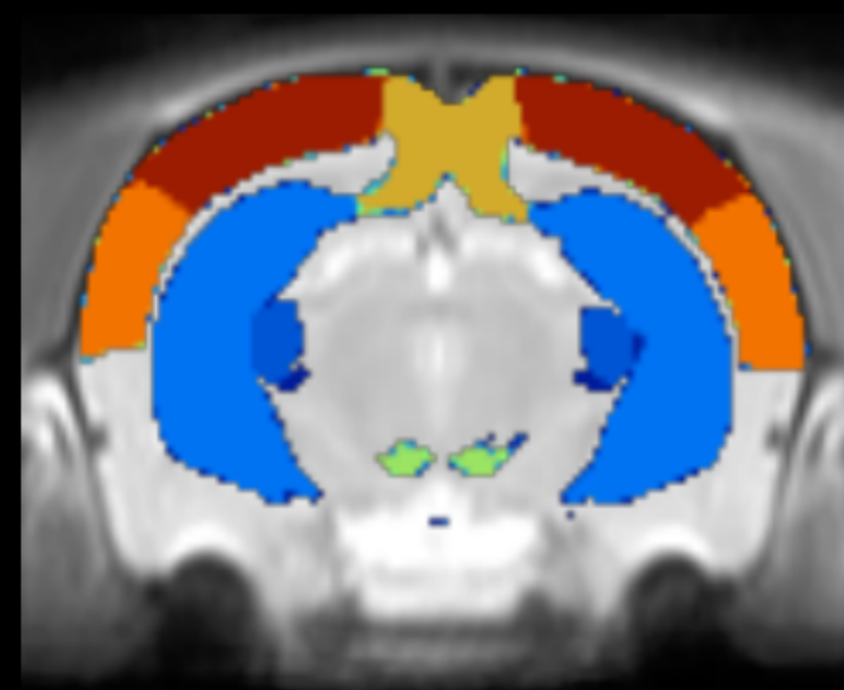

-5.1

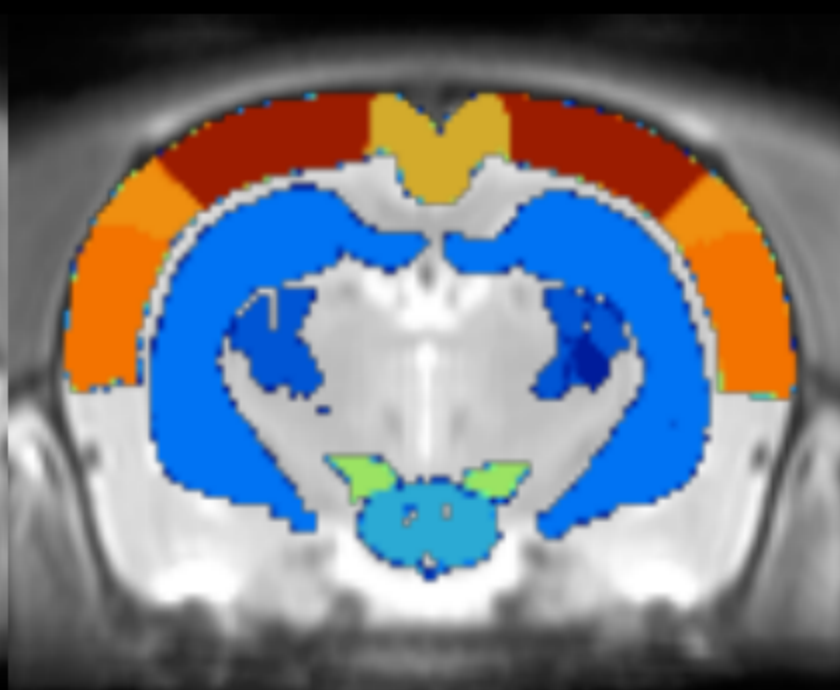

-4.1

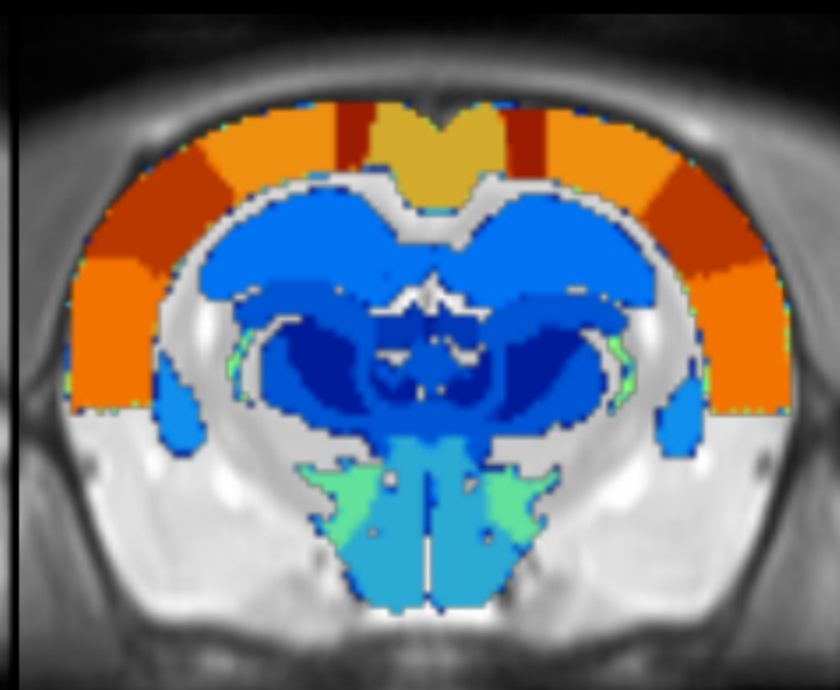

-3.1

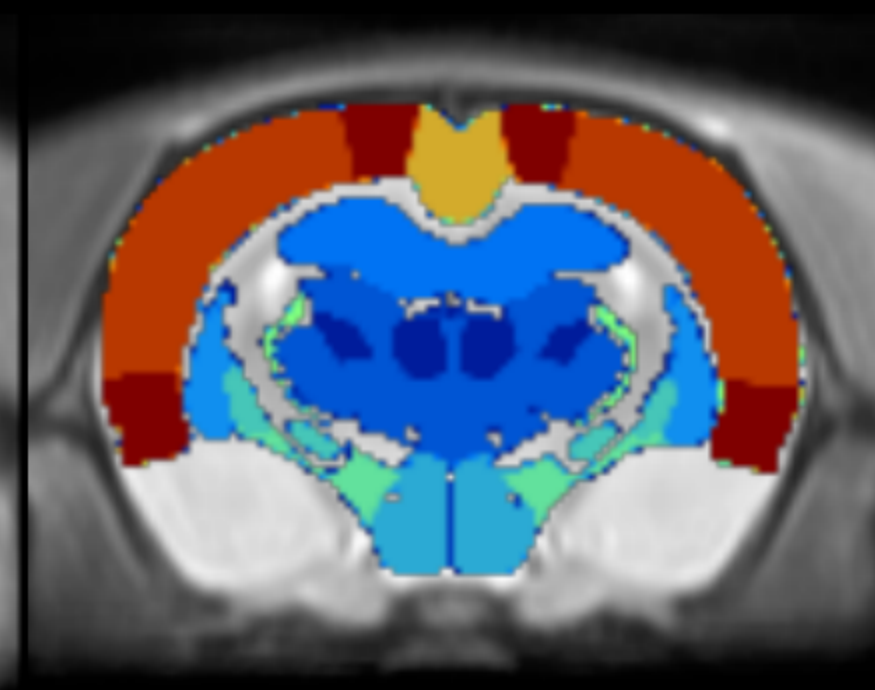

-2.1

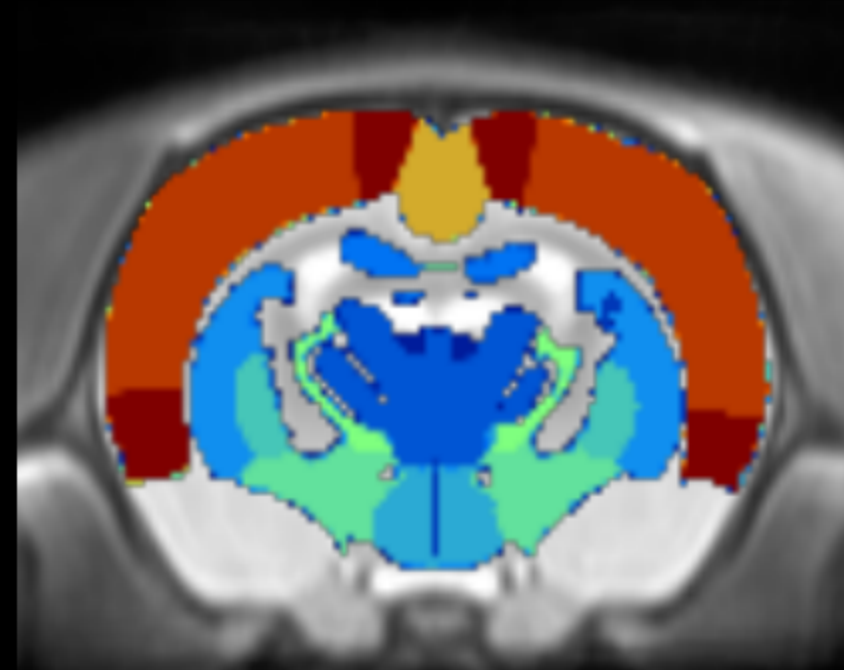

-1.1

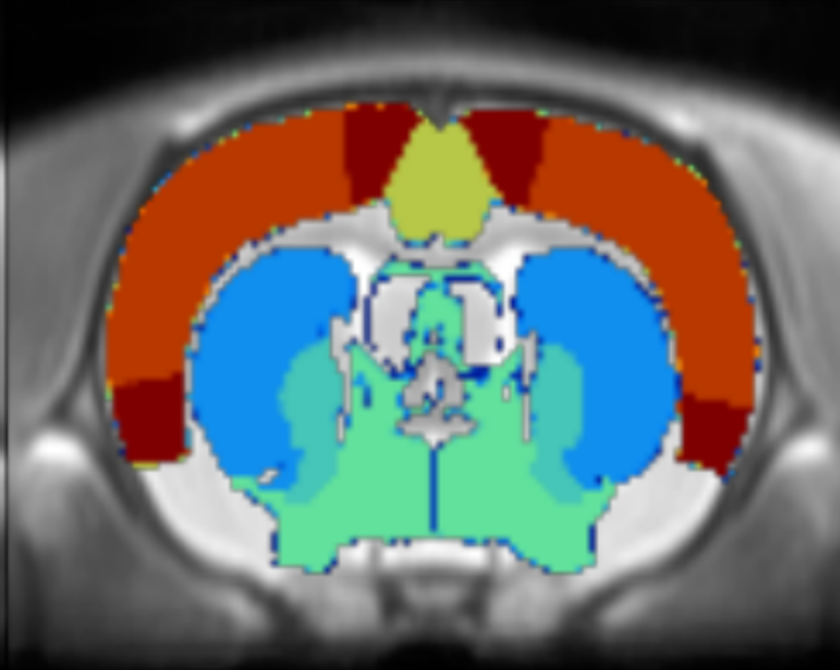

-0.1

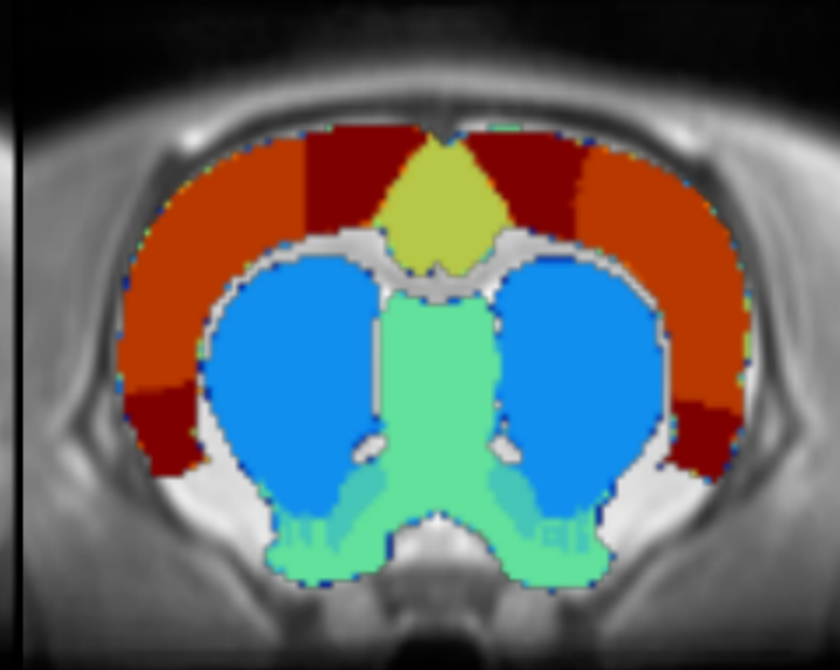

+0.9

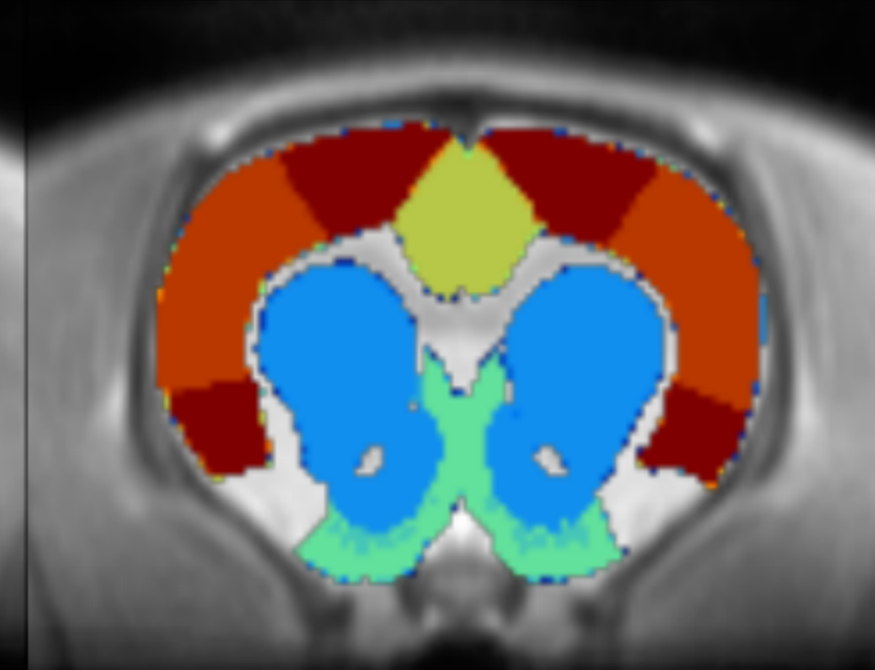

+1.9

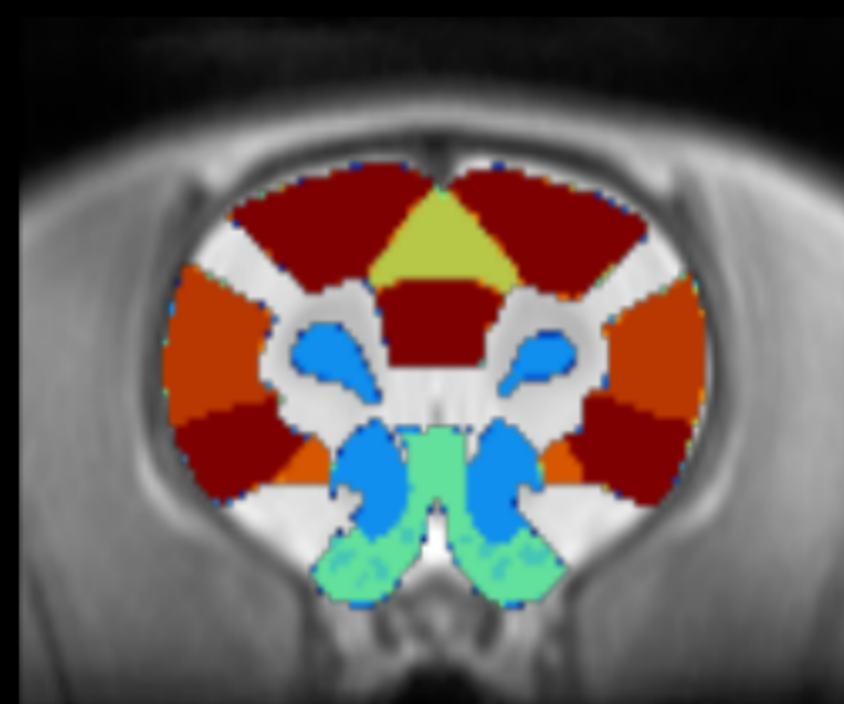

+2.9

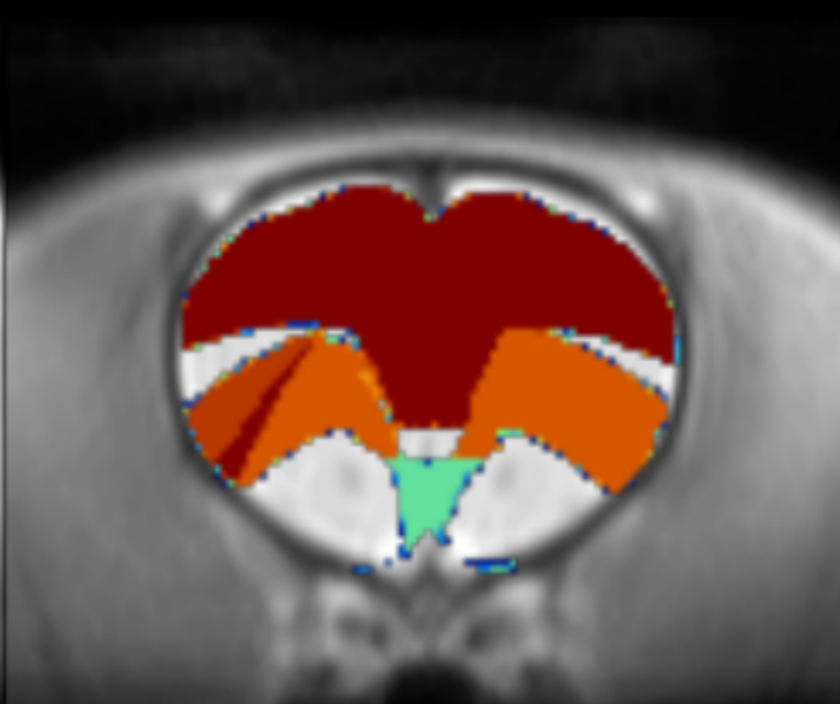

+3.9

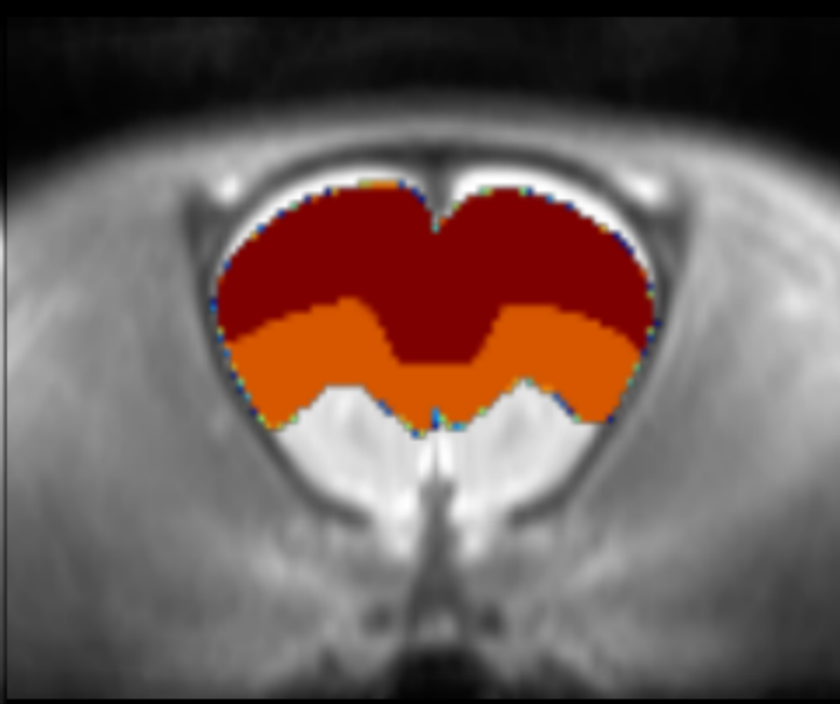

+4.9

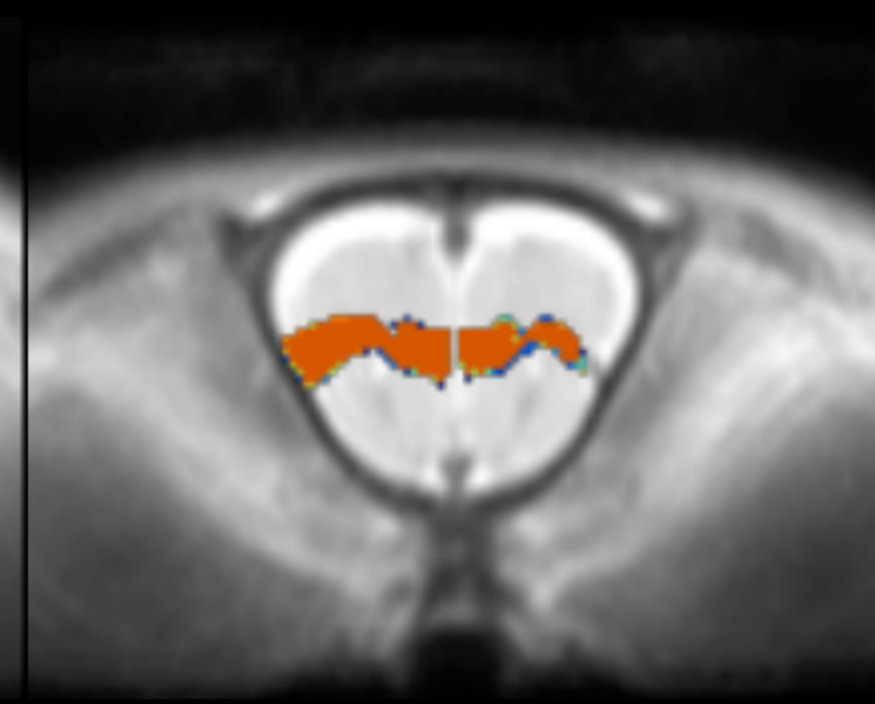

+5.9

Supplement: Supplementary file 2 — Figure S2. [file CNS-30-e14866-s004.pdf]

ketamine

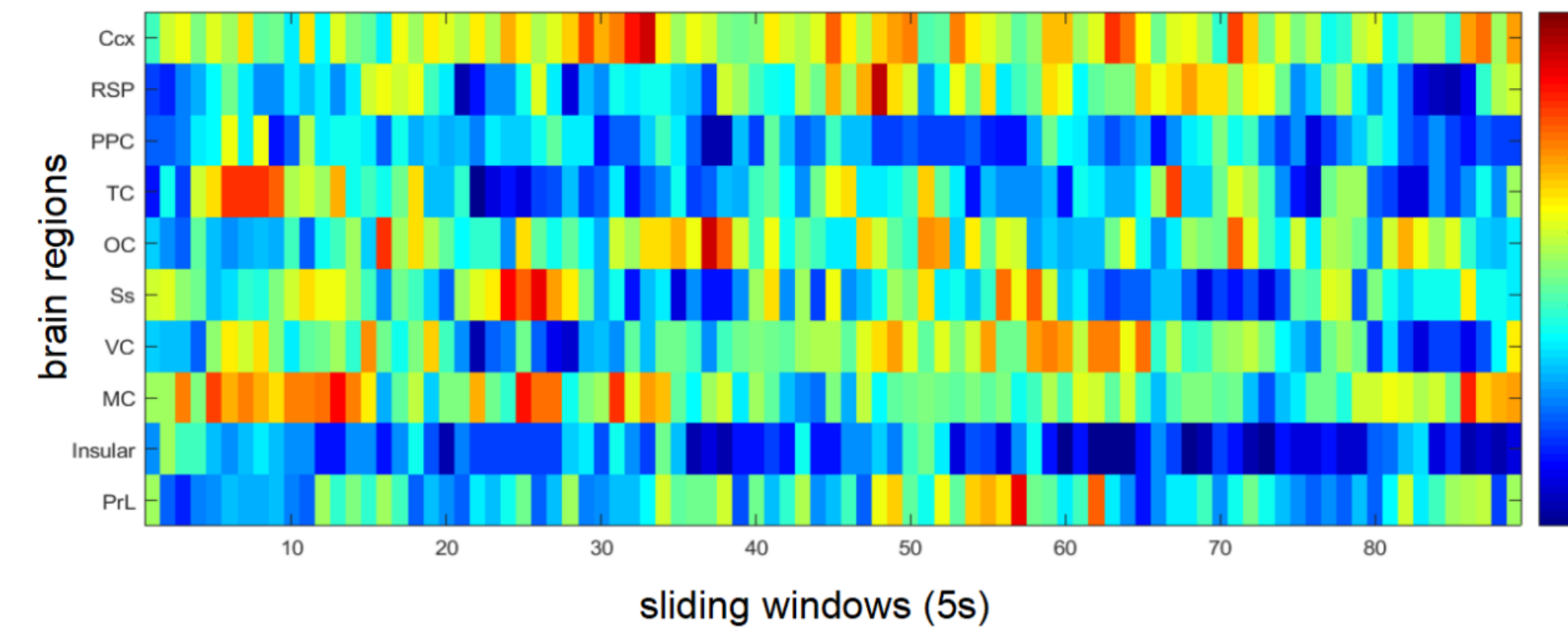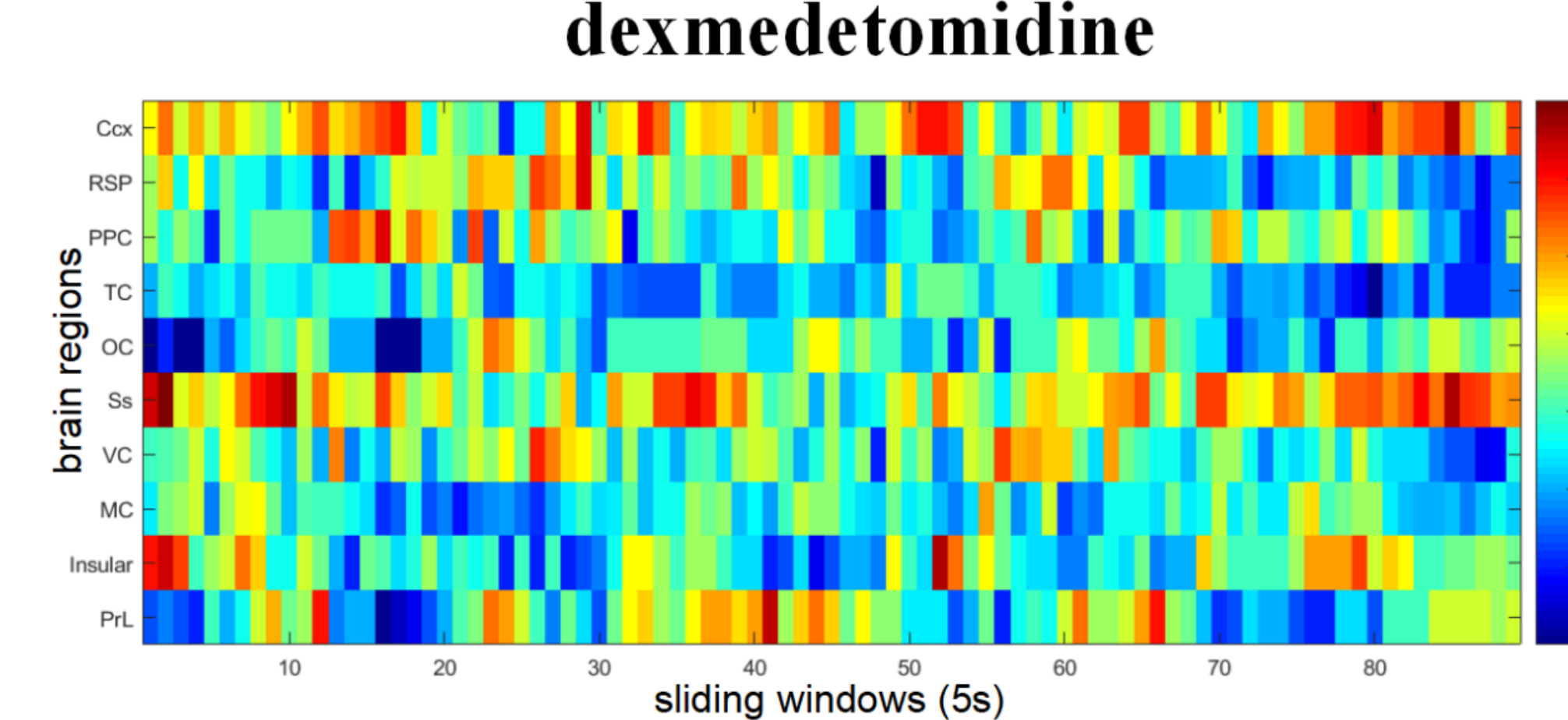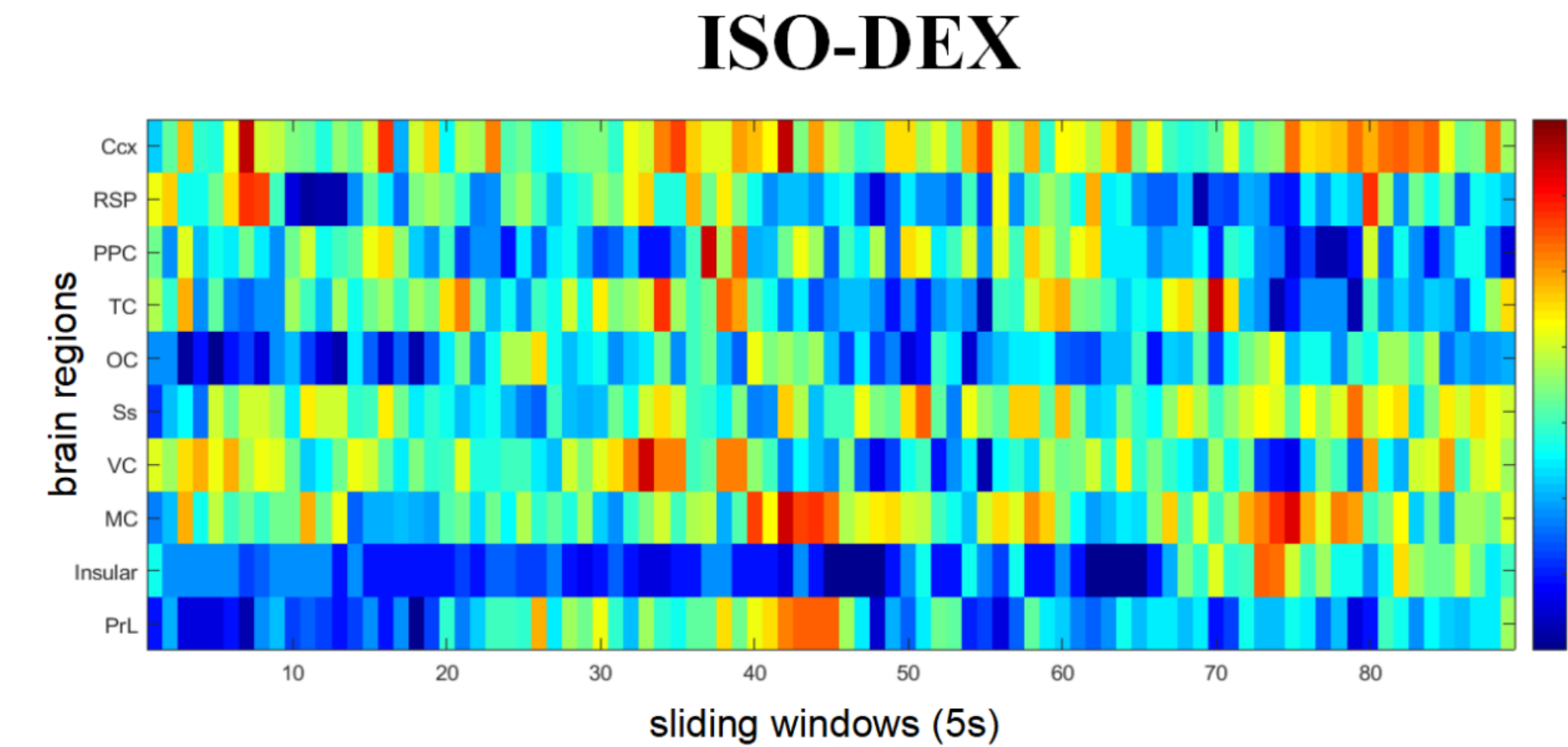

Supplement: Supplementary file 3 — Figure S3. [file CNS-30-e14866-s002.PDF]

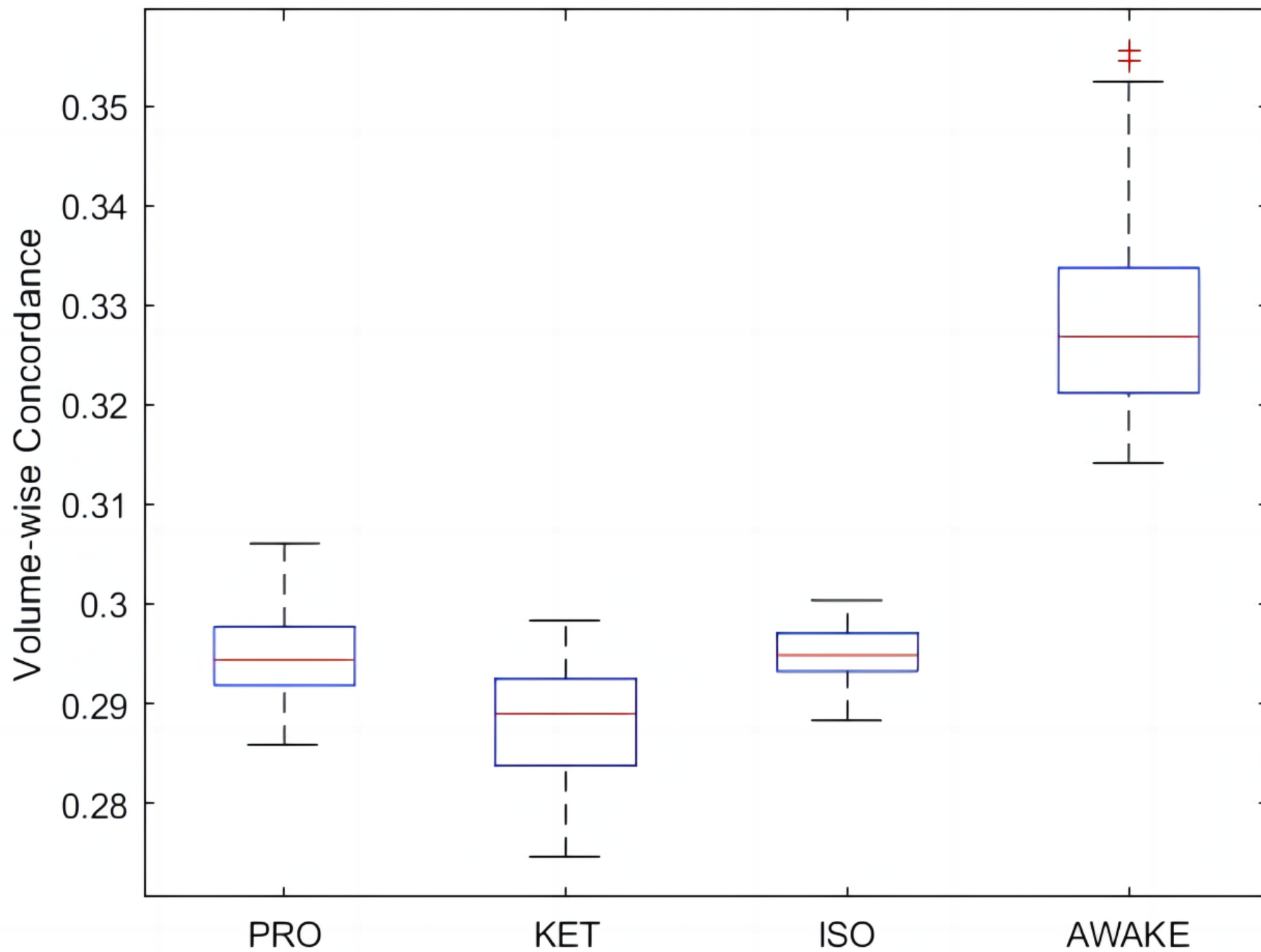

Supplement: Supplementary file 4 — Figure S4. [file CNS-30-e14866-s001.PDF]
